# Five *Pistacia* species (*P. vera*,* P. atlantica*,* P. terebinthus*,* P. khinjuk*, and *P. lentiscus*): A Review of Their Traditional Uses, Phytochemistry, and Pharmacology

**DOI:** 10.1155/2013/219815

**Published:** 2013-12-15

**Authors:** Mahbubeh Bozorgi, Zahra Memariani, Masumeh Mobli, Mohammad Hossein Salehi Surmaghi, Mohammad Reza Shams-Ardekani, Roja Rahimi

**Affiliations:** ^1^Department of Traditional Pharmacy, Faculty of Traditional Medicine, Tehran University of Medical Sciences, Tehran 1417653761, Iran; ^2^Department of Pharmacognosy, Faculty of Pharmacy, Tehran University of Medical Sciences, Tehran 1417614411, Iran

## Abstract

*Pistacia*, a genus of flowering plants from the family Anacardiaceae, contains about twenty species, among them five are more popular including *P. vera, P. atlantica, P. terebinthus, P. khinjuk,* and *P. lentiscus*. Different parts of these species have been used in traditional medicine for various purposes like tonic, aphrodisiac, antiseptic, antihypertensive and management of dental, gastrointestinal, liver, urinary tract, and respiratory tract disorders. Scientific findings also revealed the wide pharmacological activities from various parts of these species, such as antioxidant, antimicrobial, antiviral, anticholinesterase, anti-inflammatory, antinociceptive, antidiabetic, antitumor, antihyperlipidemic, antiatherosclerotic, and hepatoprotective activities and also their beneficial effects in gastrointestinal disorders. Various types of phytochemical constituents like terpenoids, phenolic compounds, fatty acids, and sterols have also been isolated and identified from different parts of *Pistacia* species. The present review summarizes comprehensive information concerning ethnomedicinal uses, phytochemistry, and pharmacological activities of the five mentioned *Pistacia* species.

## 1. Introduction

The genus *Pistacia* belongs to the Anacardiaceae, a cosmopolitan family that comprise about 70 genera and over 600 species. The species of the genus *Pistacia* are evergreen or deciduous resin-bearing shrubs and trees which are characterized as xerophytic trees and growing to 8–10 m tall. *Pistacia lentiscus* L., *P. atlantica* Desf., *P*. *terebinthus* L., *P. vera* L., and *P. khinjuk* Stocks. are distributed from the Mediterranean basin to central Asia [1, 2]. Three *Pistacia* species naturally occur in Iran: *P. vera* L., *P. khinjuk* Stocks., and *P. atlantica* Desf.; *P. atlantica* has three subspecies or varieties which have been described as *cabulica*, *kurdica,* and *mutica* [3]*. P. vera* is the only species of the genus cultivated commercially, and the rest of the species are mostly used as rootstocks for *P. vera* [1, 2].

Different parts of *Pistacia* species have been investigated for various pharmacological activities. Most of the papers are devoted to the resin of *P. lentiscus* that is known as mastic. In addition to their therapeutic effects, *Pistacia* species are used in food industry, for example, consumption of pistachio (*P. vera*) nut as food additive [4], *P. terebinthus* fruit as snack food or in making coffee-like drink [5, 6], and the anthocyanin composition of *P. lentiscus* fruit as food colorants [7].

Chemical studies on *Pistacia* genus have led to discovering diverse secondary metabolites in addition to high level of vitamins and minerals.

Our review presents a comprehensive report on phytochemical aspects, pharmacological activities, and toxicity of the genus *Pistacia* by focusing on the data reported since the year 2000 via papers on databases including PubMed, Scopus, Google Scholar, and Web of Science.

## 2. Traditional Uses

Traditional uses, plant part used, and pharmacological activities of *Pistacia lentiscus, P. atlantica*, *P. terebinthus, P. vera,* and *P. khinjuk* from different regions are listed in Table 1.

Different parts of *Pistacia* species including resin, leave, fruit, and aerial part have been traditionally used for a wide range of purposes. Among them, *P. lentiscus* is the most commonly used in different regions and resin of that has been utilized for as long as 5000 years. Resin of *P. lentiscus* has been used for variety of gastric ailments in the Mediterranean and Middle East countries for the last 3000 years [8]. It was used in ancient Egypt as incense; it has also been used as a preservative and breath sweetener [4] Most of the traditional uses reports for resin of *P. atlantica* are from Iran and have been used for the treatment of digestive, hepatic, and kidney diseases [9]. Fruit of *P. vera* (pistachio) is used all over the world. Records of the consumption of pistachio as a food date to 7000 BC [4]. Pistachio is cultivated in the Middle East, United States, and Mediterranean countries. Iran is one of the biggest producers and exporters of pistachio nuts [10]. In traditional Iranian medicine (TIM), different parts of *P. vera*, *P. atlantica, P. khinjuk P. terebinthus*, and *P. lentiscus* have been used for a long time as useful remedies for different diseases, for example, the fruit kernel of *P. vera* as a cardiac, stomach, hepatic, and brain tonic; the fruits of *P. atlantica, P. khinjuk,* and *P. terebinthus* for their aphrodisiac activity and treatment of liver, kidney, heart, and respiratory system disorders, and the gum resin of *P. lentiscus*, *P. atlantica, P. khinjuk,* and *P. terebinthus* for their wound healing activity, and treatment of brain and gastrointestinal disorders [9, 11].

## 3. Phytochemical Studies

Various compounds from different phytochemical groups were identified in *Pistacia* species. These are summarized below and also in Table 2 based on the structure of finding components.

### 3.1. Terpenoids 

#### 3.1.1. Monoterpenoids, Sesquiterpenoids, and Volatile Oil

Essential oil is one of the main components reported from different parts of *Pistacia* species including leaves, resin, ripe and unripe fruits, galls, leaf-buds, twigs, and flowers. Analysis of essential oils is mostly performed by means of gas-chromatography (GC) based techniques. There are many qualitative and quantitative variations between the content of essential oils. These variations are related to several parameters like plant species and part, sex of cultivars, harvesting time, geographical origin, and climatic conditions [12, 13]. Hydrocarbon and oxygenated monoterpens are the major chemical constituents in essential oil and among hydrocarbon monoterpens, *α*-pinene (**1**) has been reported as the main compound of some samples like *P. vera* [12, 14, 15], *P. terebinthus* [16–18], *P. lentiscus* [19–24], and *P. atlantica* [25–27]. In addition to *α*-pinene, other major components isolated from different parts of *Pistacia* species are as follows: limonene (**2**), *α*-terpinolene, and ocimene (**3,4**) from fruits and leaves of *P. vera* [28]; (*E*)-*β*-Ocimene (**5**) and limonene in fruits [18, 28, 29]; (*E*)-*β*-Ocimene and terpinen-4-ol (**6**) in leaves and *p*-cymen, (**7**) in young shoots of *P. terebinthus* [28–30]; bornyl acetate (**8**), terpinen-4-ol, sabinene (**9**), and myrcene (**10**) in fruits, terpinen-4-ol, myrcene, *p*-mentha-1 (7),8 diene (**11**), and ocimene from leaves [27, 28, 31], sabinene and *p*-mentha-1 (7),8 diene in leaf buds, and Δ^3^-carene (**12**) in unripe galls of *P. atlantica* [31, 32]. Monoterpens are also detected in mastic water which was separated from the mastic oil during steam distillation. Verbenone (**13**), *α*-terpineol (**14**), linalool (**15**), and *trans-*pinocarveol (**16**) are the main constituents of mastic water [33]. *β*-pinene (**17**) in oleoresin, *β*-myrcene and sabinene in fruits [28, 30, 34], terpinen-4-ol in aerial parts [22], and limonene, myrcene, sabinene, and teroinen-4-ol in leaves of *P. lentiscus* were determined as the main composition [28, 30, 35, 36].

Some of the other monoterpenes identified as effective antibacterial components of these essential oils are camphene (**18**), limonene, and carvacrol (**19**) from *P. vera* resin [12].

Sesquiterpenes isolated in lower amount compared with monoterpenes. Germacrene-D (**20**) and *β*-caryophyllene (**21**) were identified in *P. lentiscus* and *P. terebinthus* leaves with higher concentration in comparison with other sesquiterpenes [28]. Spathulenol (**22**), an azulenic sesquiterpene alcohol, is the predominant component of leaves of *P. atlantica* and *P. khinjuk* [37, 38]. Congiu et. al. [34] recovered Caryophyllene with the highest amount from *P. lentiscus* leaves by means of supercritical CO_2_ extraction. Germacrene-D in *P. terebinthus* flowers, *β*-caryophyllene in *P. lentiscus* galls, and Longifolene (**23**) in aerial parts of *P. lentiscus* are dominant [24, 29, 39].

#### 3.1.2. Diterpenoids

Trace amounts of Diterpenoids were isolated from the essential oil of these species. Abietadiene (**24**) and abietatriene (**25**) were detected in essential oil of *P. vera* resin [12].

#### 3.1.3. Triterpenoids

Resin of these species has been characterized by penta and tetracyclic triterpenes. Triterpenes such as masticadienonic acid (**26**), masticadienolic acid (**27**), morolic acid (**28**), oleanolic acid (**29**), ursonic acid (**30**) and their derivatives have been detected in acidic fractions of *P. lentiscus*, *P. terebinthus,* and *P. atlantica* resins [40–42]. Several triterpenoid compounds were isolated from neutral fraction of *P. lentiscus* and *P. terebinthus* resins like tirucallol (**31**), dammaradienone (**32**), *β*-Amyrin (**33**), lupeol (**34**), oleanolic aldehyde, and 28-norolean-12-en-3-one. Quantitative and qualitative varieties in chemical composition of resins according to the method of collection were reported [40, 41].

Anti-inflammatory properties have been reported from masticadienolic acid, masticadienonic acid, and morolic acid isolated from *P. terebinthus* [43]. Among triterpenes isolated from the resin of three sub-species of *P. atlantica* (*kurdica*, *cabulica* and *mutica*), 3-O-acetyl-3-epiisomasticadienolic acid (**35**) has been identified as the most effective antimicrobial agent [42].

### 3.2. Phenolic Compounds

Gallic acid (**36**), catechin (**37**), epicatechin (**38**), and gallic acid methyl ester were identified in *P. vera* seed and skin, leaves of *P. lentiscus* and leaves and galls of *P. atlantica* [44–46]. Bhouri et al. [47] demonstrated that digallic acid (**39**) from fruits of *P. lentiscus* has anti-mutagenic properties. Monounsaturated, diunsaturated, and saturated cardanols have been detected in *P. vera* kernel. 3-(8-Pentadecenyl)-phenol (**40**) was the dominating cardanol in *P. vera* [48]. Trans and cis isomers of phytoalexin, resveratrol (3,5,4′-trihydroxystilbene) (**41-42**), and trans-resveratrol-3-O-*β*-glucoside (trans-piceid) were quantified in *P. vera* kernel [49–51]. *P. lentiscus* leaf is a rich source of polyphenol compounds (7/5% of leaf dry weight) especially galloyl derivatives like mono, di, and tri-O-galloyl quinic acid (**43**) and monogalloyl glucose (**44**) [45].

1,2,3,4,6-Pentagalloyl glucose (**45**) and gallic acid from fruits of *P. lentiscus* were introduced as antioxidant and anti-mutagenic compounds [52].

Flavonoid compounds have been detected in different parts of these species. Naringenin (**46**), eriodyctyol (**47**), daizein (**48**), genistein (**49**), quercetin (**50**), kaempferol (**51**), apigenin (**52**), and luteolin (**53**) were isolated from *P. vera* fruit, and quercetin-3-O-rutinoside (**54**) is the main constituent of seed [44]. Decrease in flavonoid content of *P. vera* has been reported during the fruit ripening [51]. In addition to some known flavonoids isolated from *P. terebinthus* and *P. atlantica* fruits, 6′-hydroxyhypolaetin 3′-methyl ether (**55**) has been identified in fruits of *P. terebinthus* [46, 53]. Flavonoids were also isolated from aerial parts of *P. atlantica* and *P. lentiscus,* and quercetin-3-glucoside (**56**) was reported as the most abundant one [54]. 3-Methoxycarpachromene (**57**), a flavone with antiplasmodial activity, was isolated from aerial parts of *P. atlantica* [55].

Myricetin-3-glucoside (**58**), myricetin-3-galactoside (**59**), and myricetin-3-rutinoside (**60**) are the major flavonoid glycosides from *P. khinjuk* [54]. Myricetin derivatives also were determined as 20% of the total polyphenol amount of *P. lentiscus* leaves [45].

Anthocyanins have been reported from some *Pistacia* species. Cyanidin-3-O-glucoside (**61**), cyanidin-3-galactoside (**62**), and quercetin-3-O-rutinoside are the main anthocyanins of *P. vera* fruit [44, 56, 57]. Cyanidin-3-O-glucoside and delphinidin-3-O-glucoside (**63**) have been detected in *P. lentiscus* berries and leaves [7, 45].

### 3.3. Fatty Acids and Sterols

*Pistacia* species have oleaginous fruits considered by several researchers. The oil content in *P. vera* kernel and seed is about 50–60% [58, 59] and in ripe fruits of *P. lentiscus*, *P. terebinthus*, and *P. atlantica* is 32.8–45% [60–63]. The main fatty acid in seed and kernel of *P. vera* is oleic acid [58, 64, 65]. Oleic acid has been also determined as the most abundant fatty acid in oil of *P. atlantica* and *P. terebinthus* fruits [62, 66, 67]. Increase of oleic acid and decrease of linoleic acid have been recorded during ripening of *P. lentiscus* fruits [60]. Other fatty acids identified in these species are linolenic, palmitic, palmitoleic, stearic, myristic, eicosanoic, behenic, lignoceric, arachidonic, pentadecanoic, hexadecanoic, octadecanoic, and margaric acid [58, 66, 68].

The most abundant sterol reported in fruits of *P. vera*, *P. atlantica*, *P. lentiscus*, and *P. terebinthus* is *β*-sitosterol fallowed by campesterol, Δ^5^-avenasterol, stigmasterol, brassicasterol, and cholesterol [59, 60, 69, 70].

The oil from fruits of *P. atlantica*, *P. lentiscus,* and *P. terebinthus*, in addition to its desirable odor and taste, has been recommended as a new source for production of vegetable oils concerning the high amount of mono-unsaturated and omega-3 fatty acids like oleic acid and linolenic acid and high quantity of phytosterols like *β*-sitosterol [60, 68].

### 3.4. Miscellaneous

Chlorophylls *a* and *b* and lutein are the major colored components of *P. vera* nuts [56]. Pheophytin, *β*-carotene, neoxanthin, luteoxanthin, and violaxanthin were also determined in different samples of *P. vera* nuts [71]. *α*-tocopherol was determined in leaves of *P. lentiscus*, *P. lentiscus* var. *chia,* and *P. terebithus* [72]. Tocopherols and tocotrienols are the most abundant constituents of unsaponifiable matter of *P. atlantica* hull oil [73]. Different isomers of tocopherol, tocotrienol, and plastochromanol-8 have been identified in seed oil of *P. terebinthus* [70]. Evaluating the nutritional composition of *P. terebinthus* fruits illustrates the richness of this fruit in protein, oil, minerals, and fiber [62, 68].

## 4. Pharmacological Aspects 

Different pharmacological activities of five mentioned *Pistacia* species have been described in detail in Table 3.

### 4.1. Antioxidant Activity

Different parts and constituents from *P. lentiscus* have been shown in vitro radical scavenging properties [23, 47, 52, 74–76]. *Pistacia lentiscus* var. *chia* and *P. terebinthus* var. *chia* resins were effective in protecting human LDL from oxidation in vitro [77]. *P. atlantica* leaf and fruit have shown antioxidant activity similar to or significantly higher than those of standard antioxidant compounds in different in vitro antioxidant assays [78–80]. However, the essential oil from *P. atlantica* leaf showed weak antioxidant activity in DPPH test compared to synthetic antioxidants [32]. *P. vera* fruit revealed significant antioxidant activity similar to the synthetic antioxidant [81]. Lipophilic extract from *P. vera* nuts showed lower antioxidant potential that than of hydrophilic extract [82]. One survey showed *P. vera* skins had a better antioxidant activity compared to seeds by means of four different assays because of higher content of antioxidant phenolic compounds in skins [44]. Antioxidant activity has been also reported from other parts of *P. vera* [83].

In one study, the extract from *P. terebinthus* leaf had nearly 12-fold higher antioxidant capacity than those of BHA and ascorbic acid [84]. *P. terebinthus* fruits showed noticeable metal-chelation properties as compared to EDTA and high radical scavenging activity similar to the standards. Antioxidant activity of the fruits may be elevated by roasting process [85].

### 4.2. Antimutagenic Activity

Essential oil and different extracts from *P. lentiscus* leaves indicated significant inhibitory effect on mutagenicity in vitro [86, 87]. Gallic acid, digallic acid, and 1,2,3,4,6-pentagalloylglucose, polyphenols isolated from the fruits of *P. lentiscus*, induced an inhibitory activity against mutagenicity and genotoxicity in in vitro assays [47, 52].

### 4.3. Antimicrobial and Antiviral Activities

*Pistacia* species have demonstrated significant antibacterial activity against various Gram positive and Gram negative bacteria as shown in Table 3. Antimicrobial activity of *Pistacia lentiscus* resin, the essential oil and gum from *P. atlantica* var*. kurdica* and its major constituent *α*-pinene and *P. vera* gum against *Helicobacter pylori* were recorded [15, 33]. A study indicated that antibacterial activity of *P. lentiscus* gum oil can be attributed to combination of several components rather than to one particular compound. Verbenone, R-terpineol, and linalool showed high antibacterial activity against *Escherichia coli, Staphylococcus aureus,* and *Bacillus subtilis* which is comparable to that of mastic oil itself [19]. *P. lentiscus* gum revealed selective antibacterial activity against *Porphyromonas gingivalis* and *Prevotella melaninogenica* and had antiplaque activity on teeth by inhibiting bacterial growth in saliva [76].

Significant antifungal activity was seen from essential oil of *P. lentiscus* leaf and gum, different extracts of *P. khinjuk* leaf, and essential oil of *P. vera* gum [15, 19, 38, 88]. Evaluating the effect of *P. vera* gum essential oil on growth of 13 bacteria and 3 yeasts demonstrated inhibitory effect on all of them except *Bacillus cereus*, *Pseudomonas aeruginosa*, and *Klebsiella pneumonia* and more effective yeasticide than nystatin. Carvacrol was found to be the most effective constituent [12, 15]. Lipophylic extracts from different parts of *P. vera* showed a little antibacterial activity and noticeable antifungal one against *C. albicans* and *C. parapsilosis*. Kernel and seed extracts showed significant antiviral activity [89].

Some active constituents of essential oil from the aerial parts of *P. khinjuk* responsible for its antibacterial and antifungal activity are *α*-pinene, *β*-pinene, myrcene, beta-caryophyllene, Germacrene B, and Spathulenol [38].

Organic fraction of mastic water obtained during the steam distillation of resin from *Pistacia lentiscus* var. *chia* indicated acceptable antifungal activity but moderate antibacterial effect. Among some of its major compounds, (±)-linalool and *α*-terpineol had the highest antimicrobial effect [33].

Essential oil from leaf and gum of *P. atlantica* showed acceptable antibacterial and antifungal activities [90–92]. However, leaf ethanolic extract had no distinct antimicrobial activity [88].

A remarkable inhibitory activity of different extracts and essential oil from *P. lentiscus* leaves was observed against *Salmonella typhimurium*; additionally, essential oil showed significant inhibitory effects against *S. enteritidis* and *Staphylococcus aureus* [86, 87].

As reported by Adams et al. [55], the leaves and twigs of *P. atlantica* and its active substance 3-methoxycarpachromene showed antiprotozoal activity against *Plasmodium falciparum*. *P. atlantica* var. *kurdica* gum controlled cutaneous leishmaniasis in mice infected with *Leishmania major* [93]. Extract from *P. vera* branch had significant inhibitory activity against *Leishmania donovani* and leaf extract inhibited *Plasmodium falciparum* without cytotoxicity on mammalian cells [94].

### 4.4. Anti-Inflammatory and Antinociceptive Activity

Anti-inflammatory and antinociceptive activity of five mentioned *Pistacia* species have been shown in Table 3.

*P. terebinthus* gall showed anti-inflammatory activity in different in vivo models of acute and chronic inflammation [95]. Masticadienonic acid (**26**), masticadienolic acid (**27**), and morolic acid (**28**), three triterpene isolated from *P. terebinthus* gall, seem to be responsible for its anti-inflammatory activity [43]. Additionally, oleanonic acid (**29**) from the galls of *P. terebinthus*, reduced the production of leukotriene B4 from rat peritoneal leukocytes and showed antiedematous activity in mice [96]. Oleoresin and leaf extract from *P. vera* showed significant anti-inflammatory and antinociceptive activity [97].

Extract of the resin of *P. lentiscus* var*. Chia* and its isolated phytosterol tirucallol (**31**) showed anti-inflammatory activity on human aortic endothelial cells and had significant inhibitory activity on adhesion molecules expression in TNF-*α*-stimulated human aortic endothelial cells [98]. It was proposed that the anti-inflammatory effect of *P. lentiscus* var. *chia* gum may be related to inhibition of protein kinase C which leads to decrease in superoxide and H_2_O_2_ production by NADPH oxidase [99].

### 4.5. Effects on Gastrointestinal Disorders

One of the most important traditional uses of gums from *Pistacia* species is for management of gastrointestinal disorders. Moreover, there are several scientific studies that confirm this property [100–102]. Resin of *P. lentiscus* significantly reduced the intensity of gastric mucosal damage induced by pyloric ligation, aspirin, phenylbutazone, reserpine, and restraint with cold stress via its antisecretory and cytoprotective activities [103]. In one double-blind placebo controlled trial, *P. lentiscus* gum improved the feeling of symptoms significantly in patients with functional dyspepsia [104]. Moreover, *Pistacia* species exerted significant antibacterial activity on *Helicobacter pylori* [15, 33]. Supplementation with *P. lentiscus* oil in experimental model of colitis delayed the onset and progression of acute colitis and led to decrease weight loss caused by the disease [105]. A polyherbal formula that contains *P. lentiscus* gum caused significant decrease in colonic damage and biochemical markers related to pathophysiology of IBS in rat model of colitis [106]. Adminstration of *P. lentiscus* var. *chia* resin to patients with established mild to moderate active crohn's disease (CD) for 4 weeks caused significant reduction in CD activity index and plasma inflammatory mediators without any side effects and also as an immunomodulator resulted in significantly reduction in tumor necrosis factor-alpha (TNF-*α*) and enhanced macrophage migration inhibitory factor in these patients [107, 108].

### 4.6. Antidiabetic Activity

Aqueous leaf extract from *P. atlantica* showed significant inhibitory effect on *α*-amylase and *α*-glucosidase in vitro [109, 110]. It demonstrated significant acute postprandial antihyperglycemic activity comparable to metformin and glipizide in starch-fed rats. It also improved glucose intolerance [110]. However, another study on this extract did not show significant hypoglycemic activity when tested in normoglycemic and streptozocin-induced hyperglycemic rats [109]. Administration of *P. lentiscus* var. *chia* gum to human subjects for 12 months caused significantly decrease in serum glucose level among male subjects. Serum glucose in women was not affected [111].

### 4.7. Antitumor Activity

Among mentioned species of *Pistacia, P. lentiscus* is the most investigated for antitumor activity (Table 3). *P. lentiscus* var. *chia* gum inhibited proliferation and induced apoptosis of human colorectal tumor cells in vitro [112]. The resin exerted the most cytotoxic effect against promyelocytic leukemia among 13 human cell types and also inhibited the natural apoptosis of oral polymorphonuclear leukocytes [76]. The gum demonstrated anticancer activity via delaying the growth of colorectal tumors developed from human colon cancer cells xenografted into mice [8]. It also increased maspin (a mammary serine protease inhibitor with tumor suppressive activity for prostate cancers) expression in responsive prostate cancer cells and inhibited cell proliferation and blocked the cell cycle progression [113, 114]. Essential oil of *P. lentiscus* demonstrated significant inhibition on tumor growth in immunocompetent mice without signs of toxicity, related to apoptosis induction, reduced neovascularization, and inhibiting chemokine expression [115]. In addition, it had antiproliferative and proapoptotic effect on human leukemia cells and inhibited the release of vascular endothelial growth factor from these cells [116]. Despite many reports on antitumor activities of *P. lentiscus*, one in vivo study showed that the high dose of *P. lentiscus* gum promoted the preneoplastic lesions development in rat liver with increasing liver relative weight which proposed that desirable anticarcinogenic effects of mastic could be obtained at relatively low doses [117]. In one recent study, the current data on the anticancer activities of gum, oil, and extracts of *P. lentiscus* L. and its major constituent, have been reviewed comprehensively with special attention to the probable anticancer mechanisms [118].

The fruit extract of *P. atlantica* sub. *kurdica* showed growth inhibition in human colon carcinoma cells similar to Doxorubicin [119]. *P. vera* oleoresin demonstrated moderate cytotoxic effect against breast cancer cell line, hepatocellular carcinoma cell line, cervix cancer cell line, and normal melanocytes [120].

### 4.8. Effects on Liver and Serum Biochemical Parameters

*P. lentiscus* leaf demonstrated significant hepatoprotective activity against carbon tetrachloride induced hepatotoxicity in rats by reducing the level of bilirubin and activity of liver enzymes [121]. However, another study reported hepatic fibrosis, mild cholestasis, and depletion of reduced glutathione by long-term administration of aqueous leaf extract in healthy rats [122]. Administration of *P. lentiscus* var. *chia* gum for 18 months in healthy volunteers caused reduction in liver enzymes and exerted hypolipidemic effect [111]. Extracts from *P. vera* fruits have shown beneficial effects on HDL and LDL level in rabbit model of atherosclerosis [123]. Positive changes in lipid profile were recorded after three-week use of *P. vera* nuts in patients with moderate hypercholesterolemia. The decrease in triglyceride and LDL levels was not significant [124]. *P. terebinthus* fruit demonstrated hypolipidemic effect in hypercholesterolemic rabbits [125].

### 4.9. Effects on Atherosclerosis

More over than the antihyperlipidemic activity that described above, *Pistacia* species exerts their antiathesclerotic effects by direct activity on atherosclerotic lesions moreover than their antihyperlipidemic activity. Both methanolic and cyclohexane extracts from *P. vera* fruits have shown beneficial effects on HDL, LDL, and aortic intimal thickness in rabbit model of atherosclerosis. The methanolic extract additionally showed an antioxidant activity and remarkable decrease in aortic surface lesions [123]. *P. terebinthus* fruits inhibited the development of the atherosclerotic lesions in the thoracic artery [125]. *P. lentiscus* resin that downregulated CD36 mRNA expression (as the oxLDL receptor in macrophages that play a pivotal role in atherosclerotic foam cell formation) resulted in antiatherogenic effects [126].

### 4.10. Anticholinesterase Activity

Aqueous extracts from *P. atlantica* and *P. lentiscus* leaves showed strong acetylcholinesterase (AChE) inhibition [13]; additionally, both the methanol and ethyl acetate extracts of *P. atlantica* leaf showed relatively weak AchE inhibitory activity [127]. However, one study showed that ethyl acetate and methanol extracts of various commercially terebinth coffee brands (an oily brown-coloured powder produced from the dried and roasted fruits of *P. terebinthus*) and the unprocessed fruits of *P. terebinthus* did not have inhibitory activity against AChE and tyrosinase, while they selectively inhibited butyrylcholinesterase (BChE) at moderate levels [85].

## 5. Conclusion

In traditional Iranian medicine textbooks and papers, five species of *Pistacia* genus including *P. vera*, *P. lentiscus*, *P. terebinthus*, *P. atlantica,* and *P. khinjuk* had been introduced for treating the wide range of ailments. These species until now have been utilized in Iran by people for different nutritional and medicinal proposes. This review considered findings about phytochemical and pharmacological properties of these five species and presents comprehensive analysis of papers published since the year 2000. Ethnopharmacological data about these species may help us to know that many pharmacological aspects proposed nowadays for these species have been derived from traditional uses like antiseptic and antimicrobial, anti-inflammatory and anti-nociceptive, antihepatotoxic, and anticancer activities and their beneficial effects in gastrointestinal disorders. Furthermore, there are several pharmacological activities discussed in traditional medicine such as diuretic, lithontripic, anti-tussive, antirheumatic, antiasthmatic, antihypertensive, and aphrodisiac activities which are not supported by any current scientific documents, and so, they could be considered for investigation by researchers.

Phytochemical studies provided evidence for traditional applications of these species. With respect to phytochemical assays, triterpenes found in the resin and monoterpens are the most abundant composition of the essential oil from different parts of these species. Essential oil constituents might be valuable chemotaxonomic marker to ascertain different *Pistacia* chemotypes. Considering the therapeutic effect of isolated components, it can be concluded that terpenoids including mono, di-, and triterpenoids are associated with anti-inflammatory and antimicrobial effects. High amount of natural phenols and flavonoids is related to potent antioxidant and anticancer activities.

Review on current researches about the genus *Pistacia* L. highlighting pharmacological studies on crude plant parts, extracts, and some pure metabolites has provided scientific evidence for traditional uses and has revealed this genus to be a valuable source for medicinally important molecules.

So many studies were carried out on antioxidant activity of this genus considering their flavonoids, anthocyanins, and other phenolic compounds as preventive factors against cancer and cardiovascular diseases. *P. lentiscus* is the most studied species for antioxidant effects followed by *P. atlantica*, *P. vera*, *P. terebinthus* and *P. khinjuk*.

Most of the studies showed antimicrobial activity of these species especially *P. lentiscus* on a wide range of microorganisms including Gram-positive and -negative, aerobic and aerobic bacteria, viruses and fungi. The findings indicated that *α*-pinene, verbenone, R-terpineol, linalool, carvacrol and flavones are major compounds related to antibacrial activity.

## Figures and Tables

**Table 1:** Ethnomedicinal uses of selected *Pistacia* species.

Species	Regions	Plant part(s) used	Traditional uses and ethnobotanical reports	Reference(s)
* Pistacia lentiscus *	Algeria	Leaf	Appetizer and astringent	[75]
	Greece	Resin	Stomach ache, dyspepsia, stomach ulcer, intestinal disorders, hepatic inflammation, tooth disease, diabetes, hypercholesterolemia, and diuretic	[33, 128, 129]
		Aerial part	Stimulant, diuretic, hypertension, kidney stones, jaundice, cough, sore throat, eczema, and stomach ache	[88]
	Iraq	Resin	Abdominal pain	[130]
	Iran	Resin	Gum tissue strengthener, breath deodorizer, brain and liver tonic, and gastrointestinal ailments	[11, 100, 102]
	Italy	Leaf	Toothache, mycosis, herpes, abdominal and intestinal pain, rheumatism, antiseptic, cicatrizant, emollient, expectorant, and astringent	[131, 132]
	Jordan	Leaf	Jaundice	[121, 133]
		Resin	Heart burn and stomach ache	
	Morocco	Leaf	Digestive disease, evil eye	[134]
	Portugal	Leaf, bark	Gastric analgesic	[135]
		Root	Antiseptic and antiodontalgic	[135]
		Seeds	Antirheumatic	[135]
		Stem	Buccal antiseptic	[135]
	Spain	Aerial part	Hypertension	[136]
		Fruit	Influenza	[71]
		Leaf	Dermatophytosis in cows	[72]
		Tender bud	Warts	[73]
	Tunisia	Fruit	Edible usage, condiment, scabies, Rheumatism, and antidiarrheal	[60]
	Turkey	leaf	Eczema, diarrhea, throat infections, paralysis, kidney stones, Jaundice, asthma, stomach ache, astringent, anti-inflammatory, antipyretic, and stimulant	[96]

* Pistacia atlantica *	Algeria	Fruit	Stomach ache, cough, stress, tonic, and antidiarrheal	[20, 63]
	Greek	Fruit	Mouth flavouring, tanning, and as fodder	[31]
	Iran	Aerial part	Veterinary	[31]
		Fruit	Antidiarrheal	[11]
		Resin	Peptic ulcer, mouth freshener, antiseptic, gum tissue strengthener, as chewing gum, appetizer, phlegm dissolver, astringent, laxative, demulcent, diuretic, emmenagogue, carminative, visceral inflammation, scabies, stomach, liver and kidneys tonic, gastrointestinal disorders, and motion sickness	[9]
		Resin, bark	Joint pains, toothache, wound healing	[137]
	Jordan	Fruit	Stomach ache	[133]
		leaf	Antidiabetic	[109]
	Morocco	Leaf	Eye infection	[134]
		Resin	Gum tissue strengthener, breath deodorizer, cough, chill, and stomach disease	[27]
	Turkey	Fruit	Mouth disease	[138]
		leaf	As vegetables and food	[127]
		Resin	Wound healing	[138]

* Pistacia terebinthus *	Greece	Resin	Antidote, aphrodisiac, expectorant, and treatment of leprosy	[139]
	Iran	Resin	Smoke of it as air purifier and antiseptic	[140]
		Leaf, bark	Astringent and antidiarrhea	[11]
	Jordan	Resin	Diuretic, laxative, stimulant, and aphrodisiac	[18]
		Leaf	Diuretic, antihypertensive, and treatment of jaundice	[18]
	Spain	Aerial part	Hypotensive and cephalalgic	[141]
		Branch	Antiseptic	[141]
		Flower, leaf	Odontalgia and Dislocated joint	[142]
		Fruit	Antiprostatitis	[141]
	Turkey	Fruit	Cold, flu, diuretic, stomach ache, rheumatism, stimulant, antitussive, appetizer, as coffee, urinary inflammations, and soap production	[29, 53, 138, 143]
		Leaf	Stomach ache, mycosis, and antidiabetic	[29, 53, 144, 145]
		Resin	Urinary and respiratory antiseptic, asthma, antipyretic, and anti-inflammatory	[53]

*Pistacia vera *	Iran	Nut shell	Tonic, sedative, and antidiarrhea	[11]
		Fruit	Food	[10]
	Jordan	Oil	Facial skin cleanser	[133]
	Turkey	Resin	Asthma, stomach ache, and hemorrhoids	[146]

*Pistacia khinjuk *	Iran	Aerial part	Veterinary use	[147]
		Resin	Stomach discomfort, nausea, vomiting, and motion sickness	[148]

**Table 2:** Chemical compounds isolated from selected *Pistacia* species.

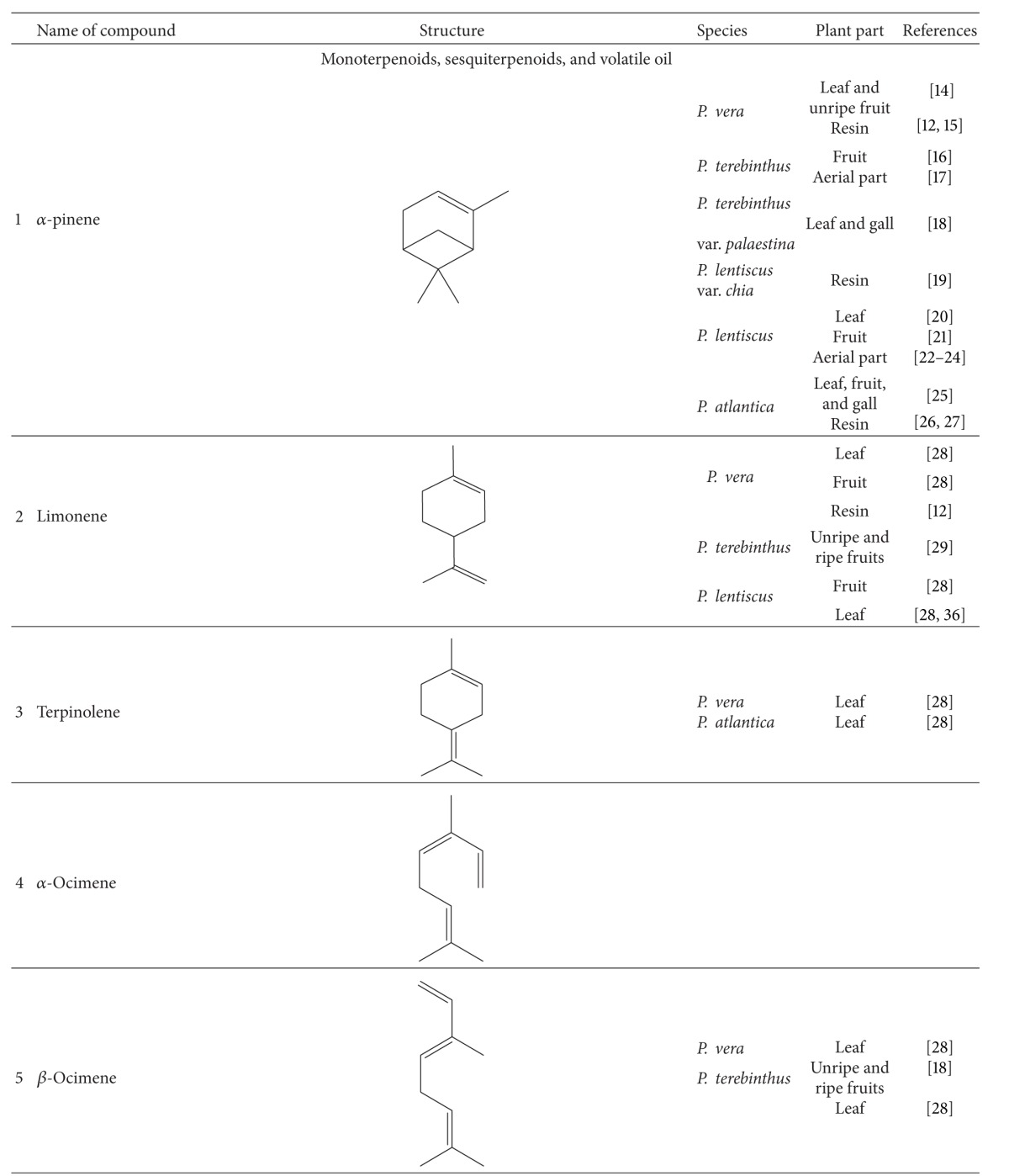 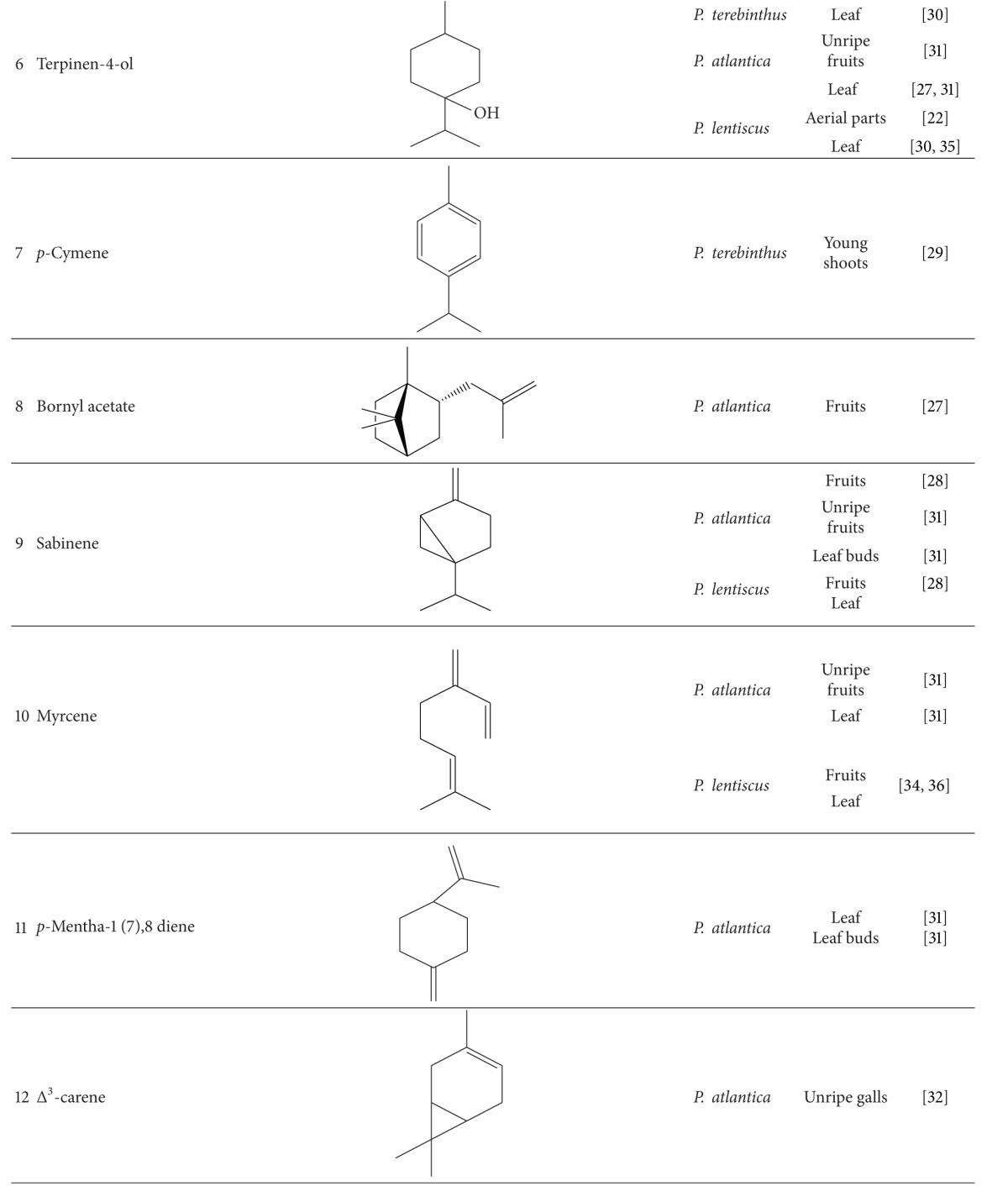 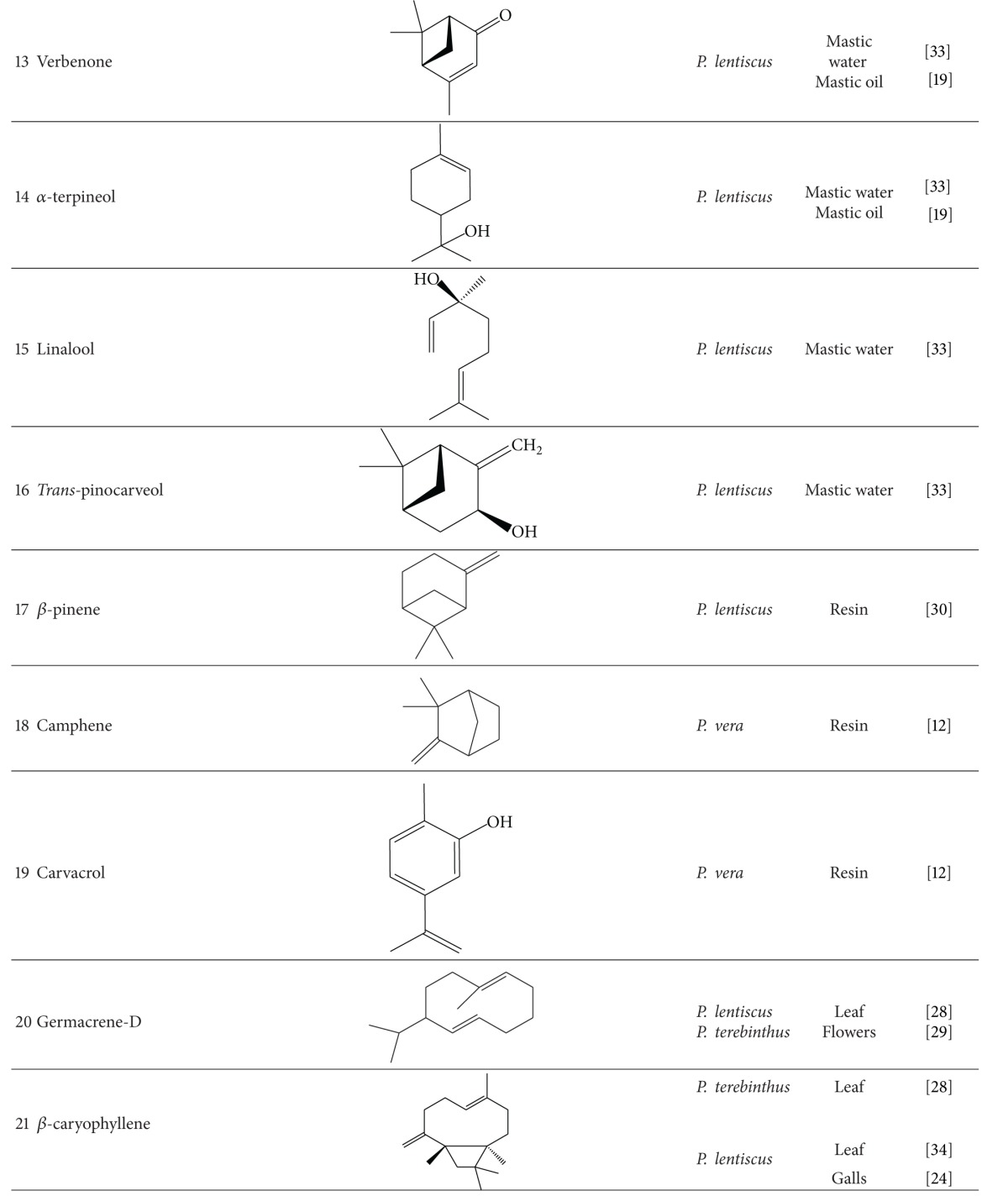 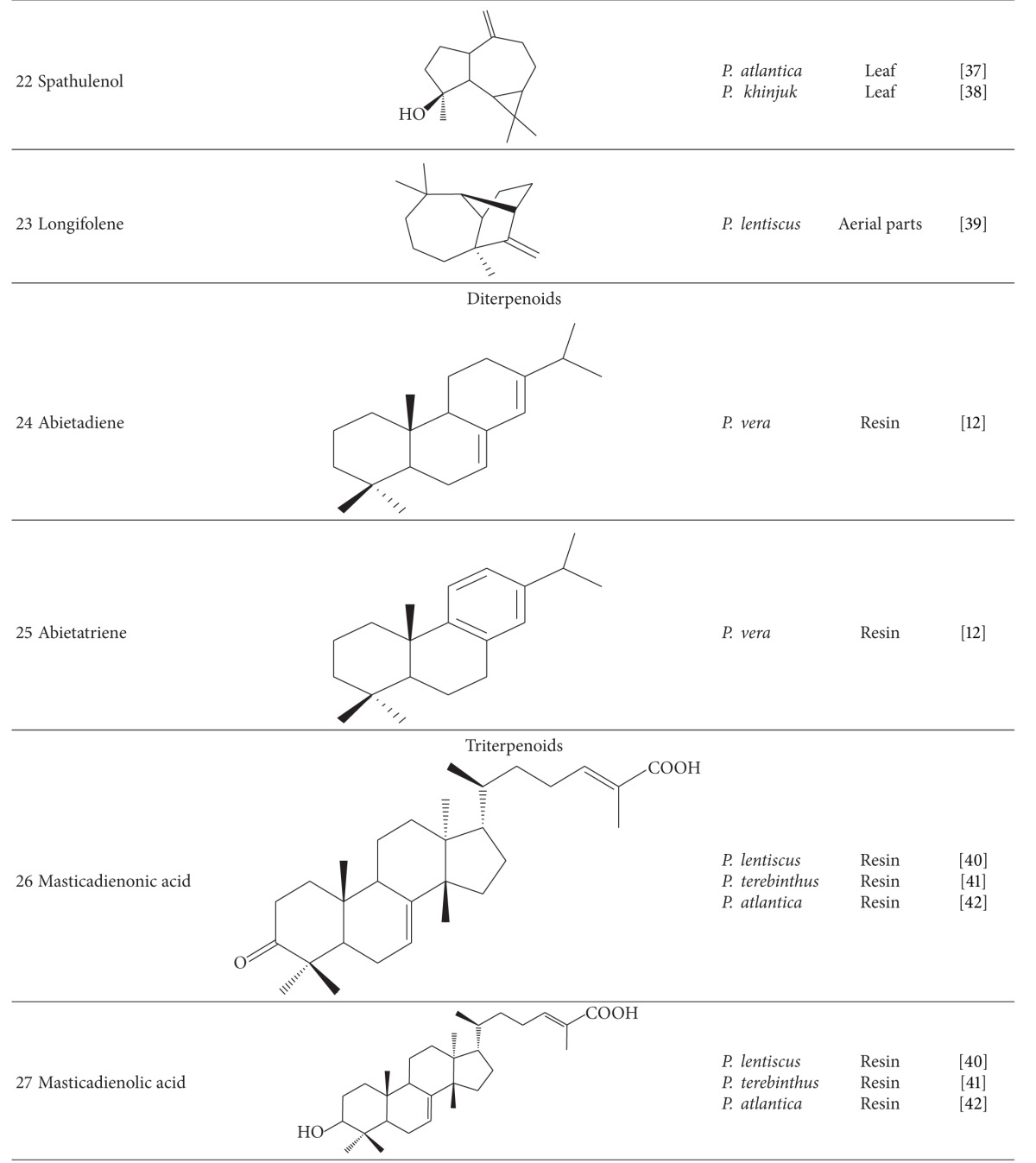 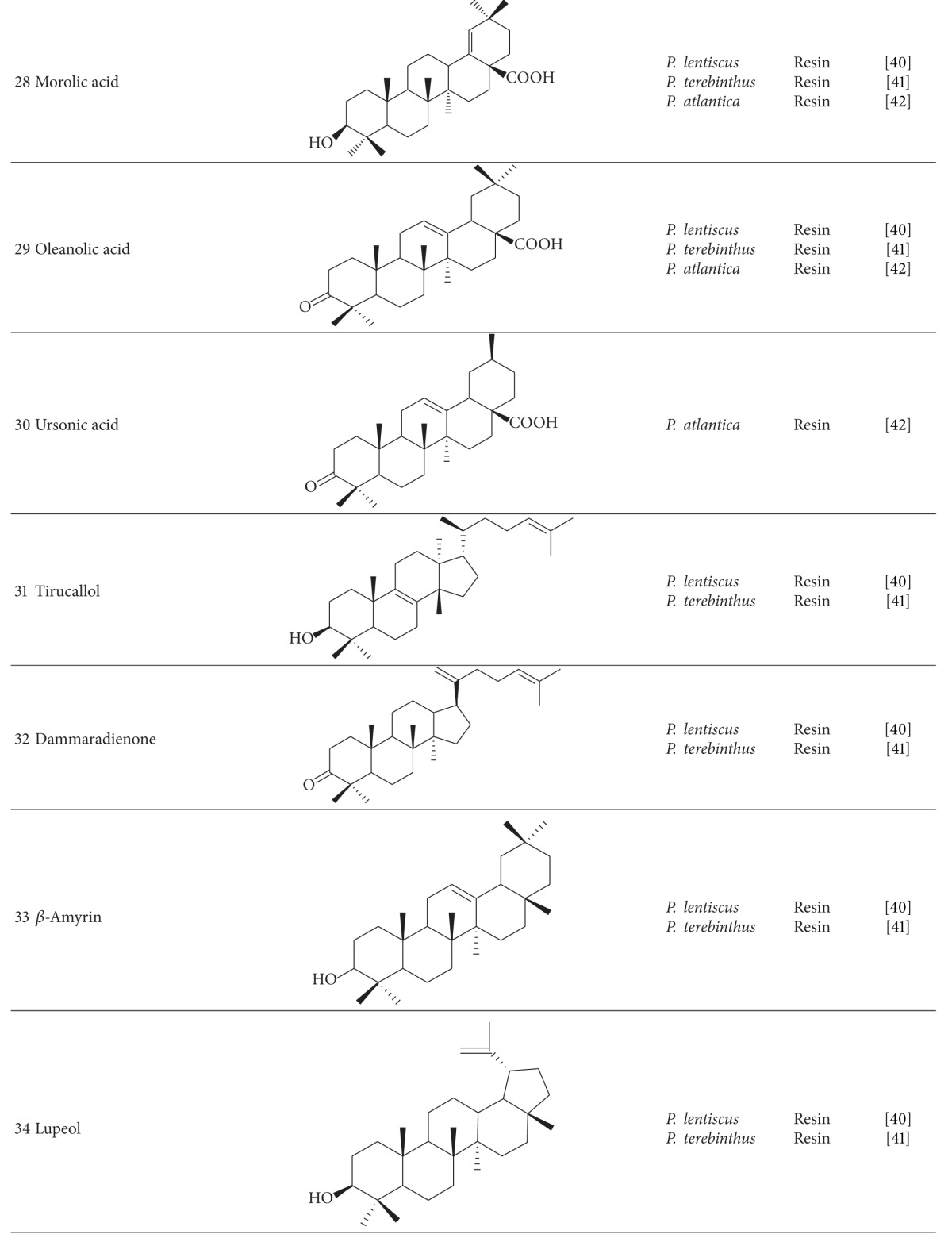 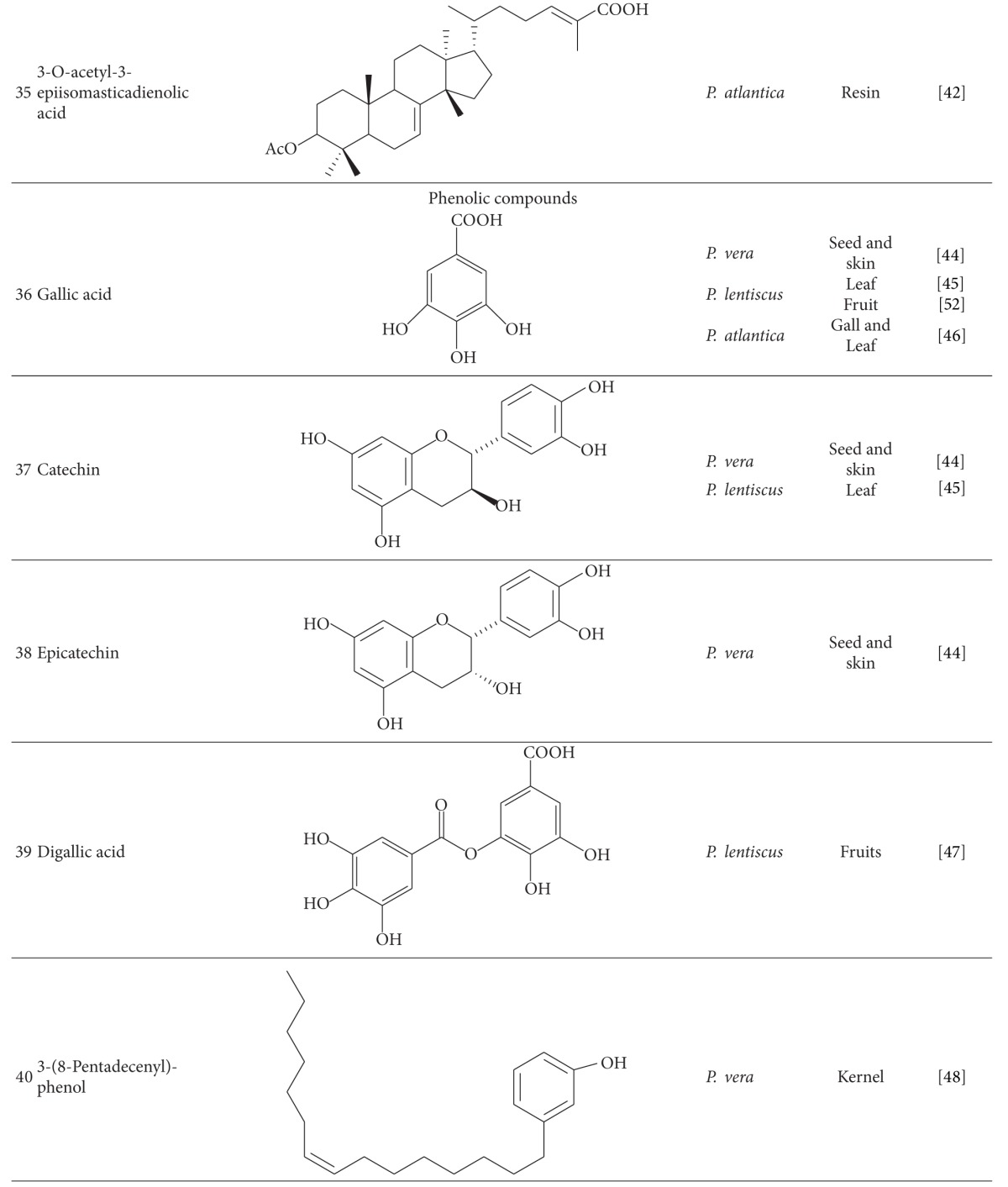 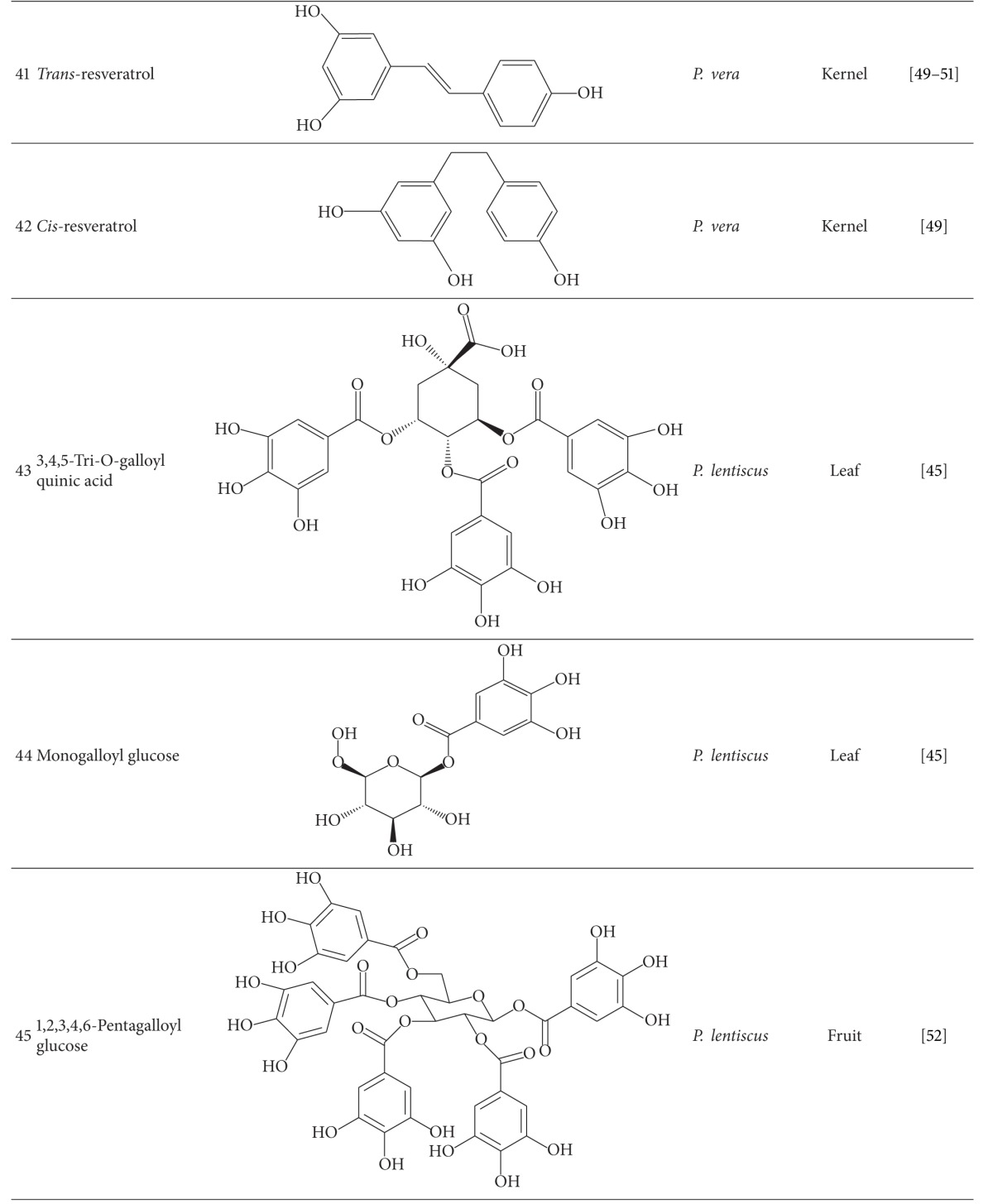 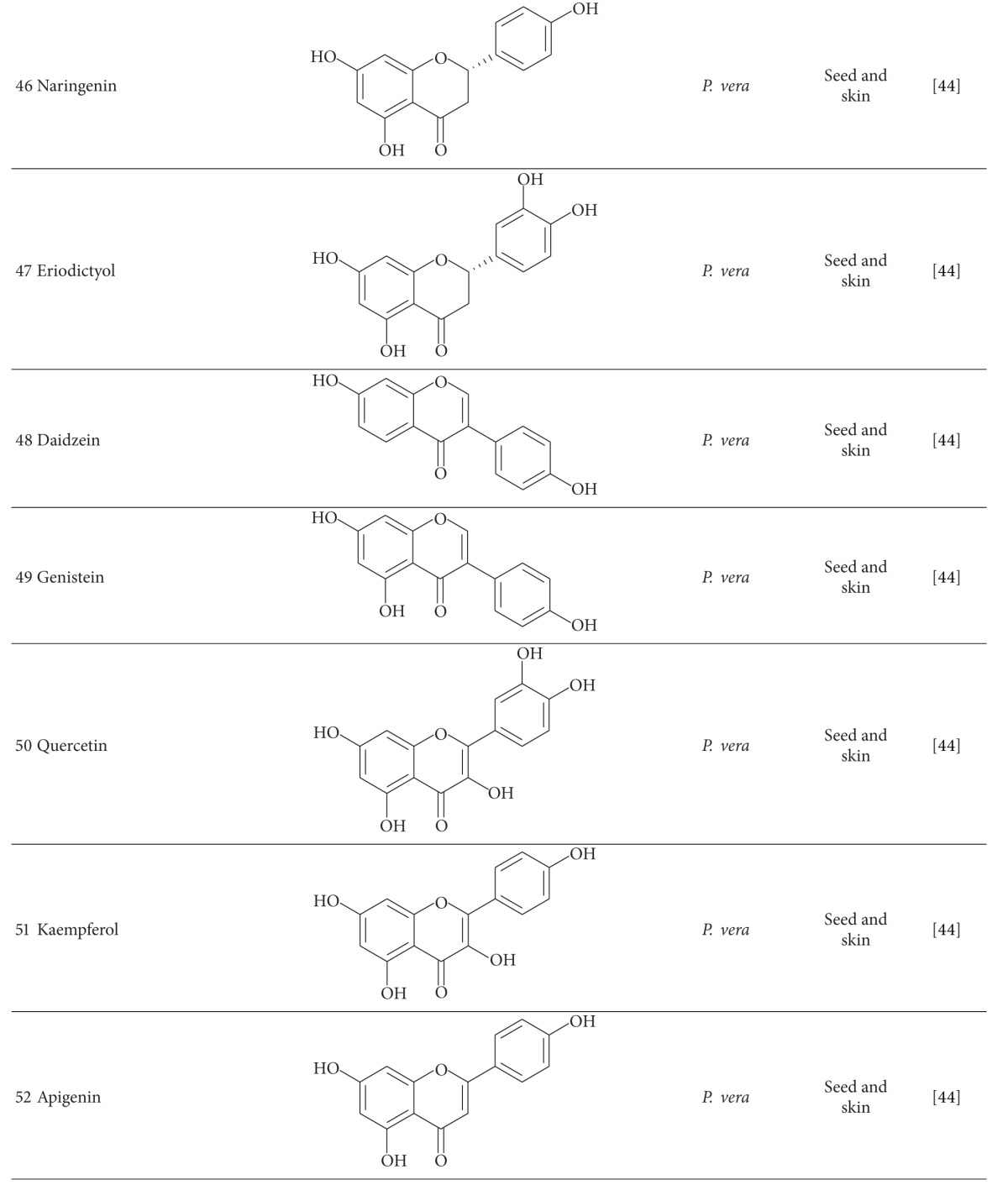 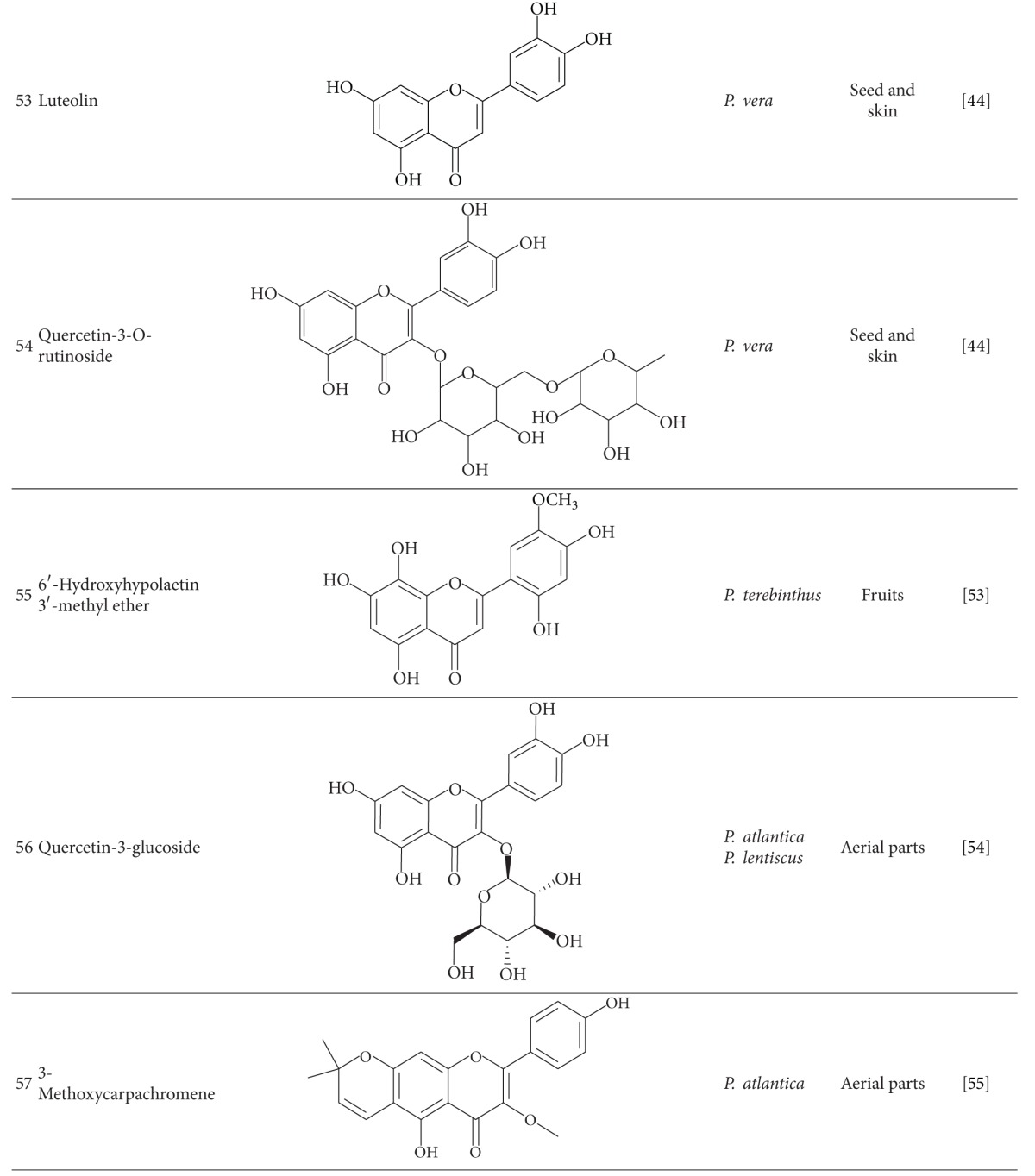 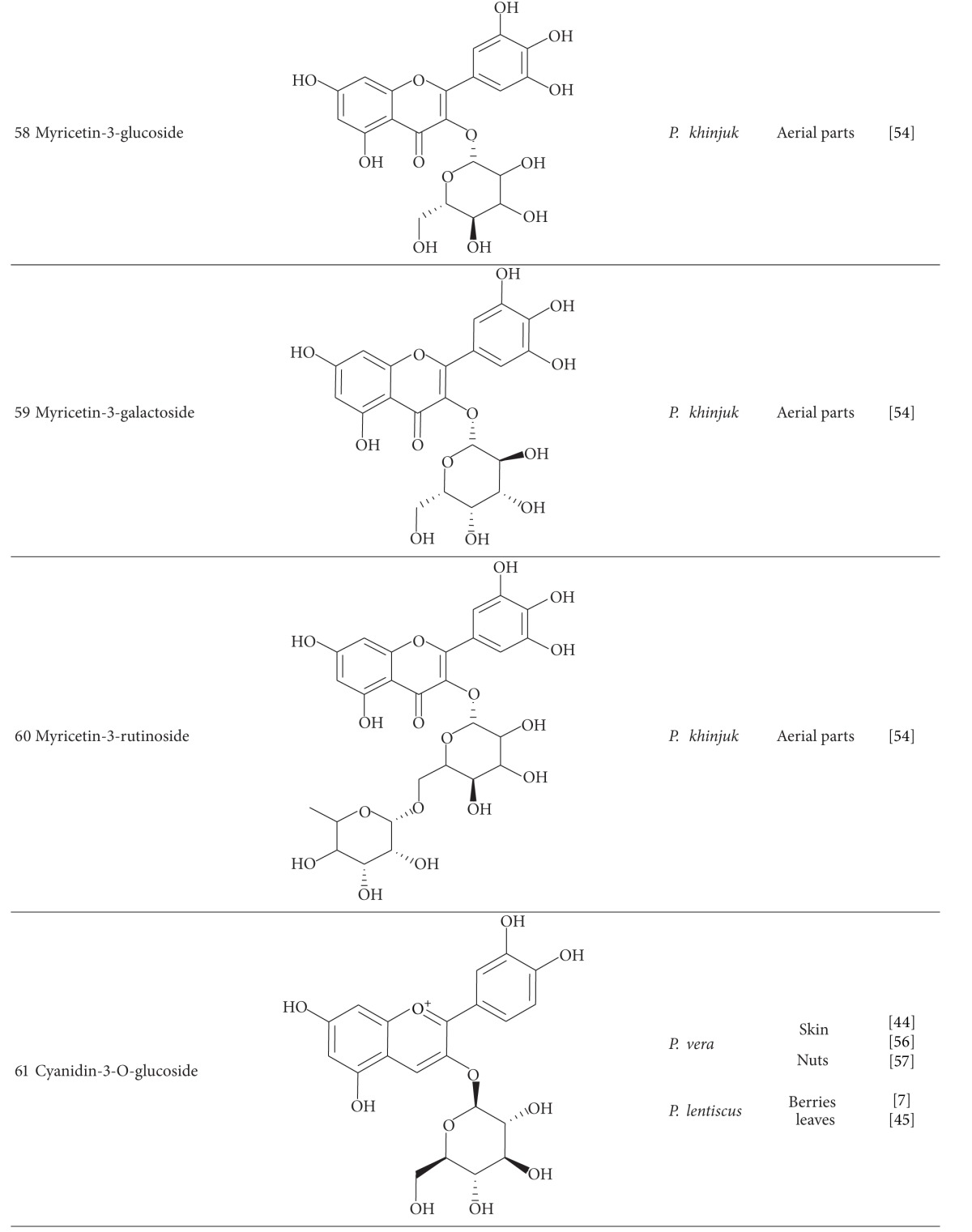 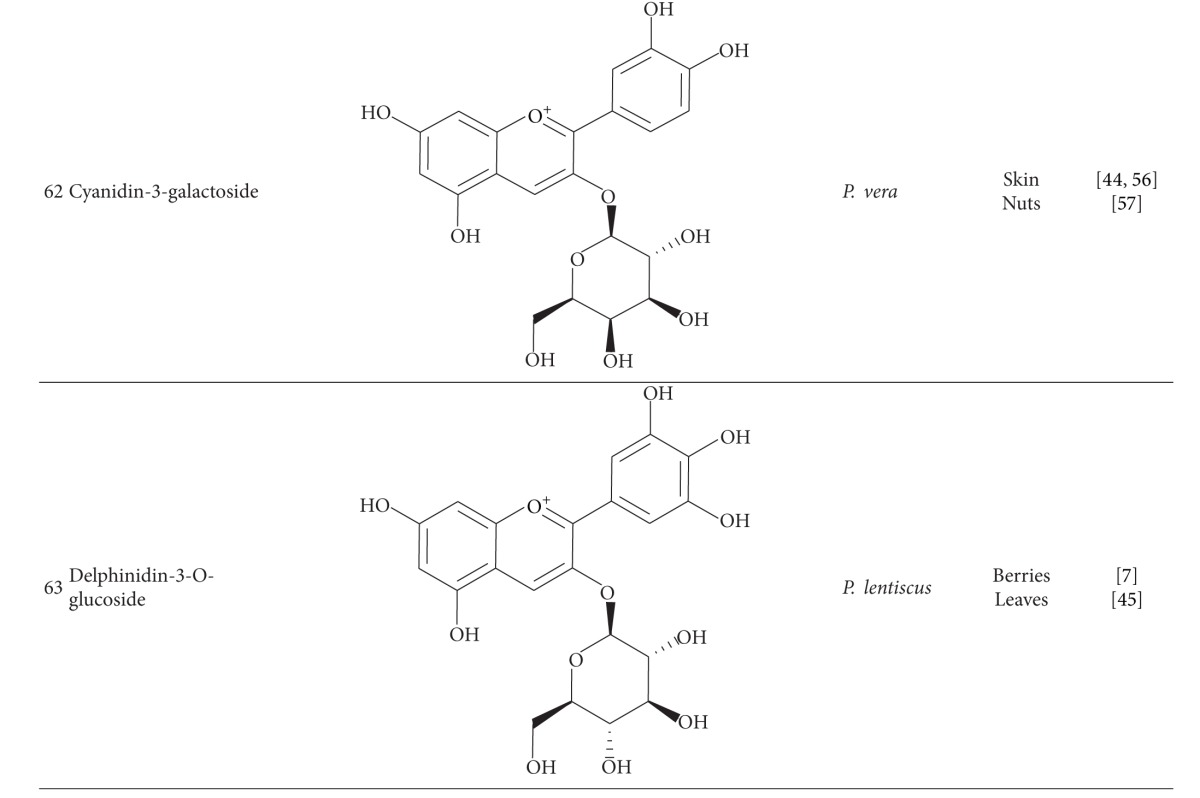

**Table 3:** Pharmacological activities of selected *Pistacia* species.

Pharmacological activity	Plant	Plant part	Assay	Extract/essential oil/isolated component	Dose or concentration	Observations	Ref.
Antioxidant	* P. lentiscus *	**Fruits	In vitro DPPH method	Polyphenols: galic acid (GA) and 1,2,3,4,6 pentagalloyl-glucose (PGA)	1, 3, 10, 30, and 100 *µ*g/mL	Dose dependent radical scavenging activity of GA (IC50: 2 *µ*g/mL) and PGA (IC50: 1 *µ*g/mL)	[52]
			Xanthine oxidase inhibition		100, 200, and 300 *µ*g/mL	↑formation of uric acid and superoxide anions (O2-) by increasing concentrations of both GA and PGA	
			Inhibition of lipid peroxidation induced by H_2_O_2_ in K562 cell line		200, 400, and 800 *µ*g/mL for GA and 100, 200, and 400 *µ*g/mL for PGA	Dose dependent inhibition by GA (IC50: 220 *µ*g/mL) and PGA (IC50: 200 *µ*g/mL)	
		**Leaf	Reducing power	Seven different extracts (1) Ethanol, (2) Ethyl acetate, (3) Aqueous/ethyl acetate, (4) Hexane, (5) Aqueous/hexane, (6) Chloroform, (7) Aqueous/chloroform	100 *µ*g/mL	Higher activity of aqueous fractions from hexane and chloroform than standards (BHA and *α*-tocopherol)	** [75]
			Linoleic acid peroxidation		100 *µ*g/mL	Inhibition of linoleic acid peroxidation by aqueous extracts from chloroform and hexane comparable to those of the standard (BHA)	
			DPPH method		10–100 *µ*g/mL	High scavenging activity (90%) equivalent to that of the standard BHA (89%) by all extracts except chloroform	
			Scavenging activity against hydrogen peroxide		100 *µ*g/mL	High scavenging capacity against H_2_O_2_ comparable to standards (*α*-tocopherol and BHA)	
		**Aerial parts	DPPH method	Essential oil	0.2, 0.4, 1.0, 2.0, and 4.0 mM	Antioxidant activity ranged between 0.52 and 4.61 mmol/L	[74]
			DPPH method	Methanolic extracts	100, 80, 50, 30, 20, 10, and 5 mg/L	IC50 ranged between 5.09 and 11.0 mg/L	**[23]
			FRAP assay		5000 mg/L	Activity ranged between 84.6 and 131.4 mmol Fe2+/L plant extract; IC50: 5.09–11.0 (mg/L)	
	*P. lentiscus* var*. chia *	Resin	Oil oxidation assay by the oven test	Resin solution in dichloromethane	0.05, 0.1, and 0.15% w/w	Significant antioxidant activity	[149]
	* P. lentiscus *	Fruit	ABTS	Digallic acid	0.05, 0.1, 0.15, and 0.2 mg/mL	Free radical scavenging activity towards the ABTS + radical was 99% at 0.2 mg/mL	**[47]
			Xanthine oxidase (XO) inhibition and superoxide scavenging activity		50, 100, and 150 *µ*g/mL	21% XO inhibitory activity at 150 *µ*g/mL; 28% reduction of superoxide anion activity	
			TBARs		200, 400, and 800 *µ*g/mL	↓lipid peroxidation (IC50: 178 *µ*g/mL)	
		Gum	Electron-spin resonance Spectroscopy for the determination of hydroxyl radical by Fenton reaction	Mastic in water	ND	Effectively scavenged hydroxyl radical generated by the Fenton reaction	[76]
			Nitrate/nitrite colorimetric assay		0–3 mg/mL	No nitric oxide scavenging activity	
	*P. lentiscus* var*. chia, P. terebinthus.* var*. chia *	Gum	Copper-induced LDL oxidation	Hexane and methanol/water extracts	2.5, 5, 10, 25, and 50 mg/2 mL	LDL protective activity; methanol/water extract of *P. lentiscus* showed the most LDL protection	[77]
	*P. lentiscus *	Leaf	Reduction power activity	Ethanolic extract	0.25; 0.5; 0.75; 1; 2; 3 mg/mL	Reducing power comparable to ascorbic acid	[88]
			Pyrogallol autoxidation method		ND	Superoxide anions scavenging activity	
	*P. atlantica. *	Leaf	Reduction power activity	Ethanolic extracts	0.25; 0.5; 0.75; 1; 2; 3 mg/mL	Reducing power close to values observed by ascorbic acid	[88]
			Pyrogallol autoxidation method		ND	Superoxide anions scavenger at a concentration as low as 0.0625 mg/mL	
	*P. atlantica* subsp*. mutica *	Hull	FRAP test	The unsaponifiable matter (USM) of fruit's hull oil	100 mg in 10 mL of *n*-hexane	Significant reducing power; the highest reducing power amongst the USM fractions belonged to the tocopherols and tocotrienols and linear and triterpenic alcohols respectively	[80]
			DPPH radical-scavenging assay		ND	EC50 value significantly lower than *α*-tocopherol	
			Oven test		ND	Significant stabilizing effect	
	*P. atlantica *	Leaf	(1) Reducing power (2) Chelating abilities on metallic ions (3) Radical scavenging Activity (DPPH) (4) The total antioxidant activity (thiocyanate method in linoleic acid emulsion) (5) Hydrogen peroxide scavenging activity	Decoction	(1) 20–100 *μ*g/mL (2) 0.25, 0.50, 0.75, and 1.0 mg/mL (3) 5–25 *μ*g/mL (4) 100 *μ*g/mL (5) 100 *μ*g/mL	(1) Reducing power of significantly higher than *α*-tocopherol and BHT and nearly similar to BHA (2) The chelating activity of 1.0 mg/mL was nearly fourfold less than EDTA at 0.037 mg/mL and has slightly effective capacity for iron binding (3) 85% inhibition rate at 15 *μ*g/mL. nearly similar to ascorbic acid and BHA (4) Higher antioxidant activity than *α*-tocopherol and similar to BHA, BHT, and trolox (5) Concentration-dependent scavenging compared to BHA, BHT, and *α*-tocopherol	[78]
	*P. atlantica* subsp*. mutica *	Fruit hull	Rancimat test	*n-*Hexane extract	Different percentages (up to 15%)	The antioxidant activity of hull oil was exactly the same as that of TBHQ at low concentrations	[79]
	*P. atlantica *	Leaf	DPPH test	Essential oil	50 *µ*L	Weak radical scavenging activity	[32]
			FRAP test		ND	Higher antioxidant capacity relative to ascorbic acid	
	*P. vera *	Fruit hull	Oven test	Water and methanol extracts	0.02%, 0.04%, and 0.06% in soybean oil	Effective in retarding oil deterioration at 60°C; at concentration of 0.06%, similar to BHA and BHT added at 0.02%.	[81]
	*P. vera* L*., *var.* Bronte *	Kernel	ABTS radical cation decolorization assay	Methanol/water or Dichloromethane	ND	The antioxidant activity of the lipophilic extract was much lower than hydrophilic one	**[82]
			Lipid peroxidation (TBARS assay)	Hydrophilic extract	0.25, 0.5, or 1.0 mg/mL	Radical scavenging activity in a dose-dependent manner	
			Copper-mediated LDL oxidation	Hydrophilic extract	Extracts from 30, 60, or 100 **µ**g of nut	Inhibition of LDL oxidation	
		Seed and skin (hull)	DPPH assay	Methanol/water extract	0.050–12.00 mg/mL	Radical scavenging activity	**[44]
			Trolox equivalent antioxidant capacity (TEAC) assay (ABTS radical)		ND	Antioxidant power: 0.015 ± 0.001 and 2.19 ± 0.14 mmol Trolox/g of seeds and skins, respectively	
			Scavenging activity against the superoxide anion		ND	IC50 of 3.25 ± 0.19 and 0.25 ± 0.02 mg for seeds and skins, respectively	
	*P. vera *	Gum	TBARS and FRAP in rat	Extract	0.1–0.5 g/kg	↓brain MDA level by 63% and ↑antioxidant power of brain by 235%	[83]
		Hull	DPPH assay	Aqueous	1, 1.5, 2,5, 3,5 and 4 *μ*g/mL	Concentration-dependent radical scavenging activity	[150]
			ABTS assay		ND	Scavenging capacity of crude and purified extracts was higher than standards compounds (TBHQ and BHT)	
			*β*-carotene bleaching method		0.48–9.5 *μ*g/mL	Concentration-dependent antioxidant capacity	
	*P. terebinthus *	Leaf	Trolox equivalent antioxidant capacity assay (ABTS/K2S8O2 method)	Ethanol-water extract	ND	Considerably higher antioxidant activity compared with BHA and ascorbic acid	[84]
		Fruits	DPPH test	Acetone and methanol extracts	25, 50 and 100 *µ*g/mL	High radical scavenging activity	[53]
			Total antioxidant activity in *β*-carotene-linoleic acid system		25, 50 and 100 *µ*g	Isolated pure 60-hydroxyhypolaetin-30-methyl Ether showed higher antioxidant activity than both extracts and BHT	
			Superoxide anion scavenging activity		50 *µ*g	Both extracts had scavenging activity near to ascorbic acid; higher activity of methanol extract than acetone extract	
			FRAP		0.2–1 *µ*g/mL	Higher reducing power of methanol extract than *α*-tocopherol; acetone extract reducing power was equal to that of *α*-tocopherol	
			Metal chelating activity		1000–4000 *µ*g/mL	Methanol extract had higher activity than acetone extract	
		Fruits and 4 terebinth coffee brands	DPPH radical scavenging activity	Ethyl acetate and methanol extracts	250, 500, 1000 and 2000 *µ*g/mL	High scavenging effect especially at 2000 *μ*g/mL	[85]
			DMPD radical scavenging activity			Scavenging effect lower than that of quercetin	
			H_2_O_2_ radical scavenging activity			Inactive in scavenging H_2_O_2_ radical	
			Metal-chelation effect			Remarkable metal-chelation properties as compared to EDTA	
			FRAP assay			High reducing power	
			PRAP assay			High reducing power	

Antimutagenic	* P. lentiscus *	Leaf	Aflatoxin B1 (AFB1)-induced mutagenicity in *S. typhimurium* TA 100	Essential oil	250, 500 and 1000 *µ*g/plate	Mutagenic inhibition of 76.7% by 250, 82.8% by 500, and 96.5% by 1000 *µ*g/plate	[86]
			(AFB1)-induced mutagenicity in *S. typhimurium* TA100 or TA98	Essential oil	0.3, 250, 500, 1000 *µ*g/plate	In TA100: 76, 82.8, and 96.5%, mutagenic inhibition rate for 250, 500, and 1000 *µ*g/plate, respectively; in TA98: 99 and 100% mutagenic inhibition rate with 250 and 500 *µ*g/plate	[87]
				Aqueous extract	0.3, 50, 300, 600 *µ*g/plate	50 *µ*g/plate: 23% inhibition in TA100 and 52.2% in TA98; 300 and 600 *µ*g/plate: 67.7 and 87.8% for TA100 and 58–76.8% for TA98	
				Flavonoid-enriched extract extracts	50, 300, 600 *µ*g/plate	TA100: 47, 75.3, and 88.6% inhibition by 50, 300, and 600 *µ*g/plate, respectively; TA98: 62.5, 77, and 93.5% inhibition by 50, 300, and 600 *µ*g/plate, respectively	
			Sodium azide- induced mutagenicity in *S. typhimurium* TA1535 and TA100	Essential oil	1.5, 10, 15, 30 *µ*g/Plate	TA100: 79, 83, and 94% inhibition by 10, 15, and 30 *µ*g/plate, respectively; TA1535:, 62, 76, and 93% inhibition by 10, 15, and 30 *µ*g/plate, respectively	
				Aqueous extract	1.5, 50, 300, 600 *µ*g/plate	TA100: 92, 96, and 98% inhibition by 50, 300, and 600 *µ*g, respectively; TA 1535: 62, 80, and 94% for the same concentrations	
				Flavonoid-enriched extract extracts	50, 300, 600 *µ*g/plate	50 and 300 *µ*g/plate: from 54 to 68% inhibition in TA1535 and from 84 to 93% in TA100	

Anitmicrobial and antiviral	* P. lentiscus *	**Leaf	Disc diffusion	Essential oil	0.03, 0.15, 0.62, 2.5, 10.0, 40.0 mg/mL	Noticeable activity against *S. enteritidis *(MIC: 30 *µ*g/mL) and *St. aureus* (30 *µ*g/mL); less important activity against *S. typhimurium*, (MIC: 150 *µ*g/mL); No significant inhibitory activity towards *Escherichia coli*, *Pseudomonas aeruginosa*, and *Enterococcus faecalis *	[86]
			Disc diffusion	Ethanolic extract	5 and 10 *μ*L	No effect on *Klebsiella pneumoniae *and *Escherichia coli. *Significant inhibition against* Candida albicans, Staphylococcus aureus*, and *Salmonella typhi *	[88]
			Disc diffusion	Ethanolic extract	50, 100, 500 *μ*L, and 1 mL	Inhibiting activity on *Trichoderma sp* and *Fusarium sp *	[88]
			Disc diffusion	Aqueous extract	ND	Most active against *S. typhimurium*, (MIC: 4 *μ*g/mL), significant inhibitory activity towards *P. aeruginosa* and *S. enteritidis* (MIC: 40 *μ*g/mL), and no activity against *S. aureus*, *E. coli*, and *Ent. faecalis* up to 1000 *μ*g/mL	**[87]
			Disc diffusion	Total oligomer flavonoid-enriched extract	ND	TOF extract exhibited antibacterial activity only against *S. typhimurium* (MIC: 100 *μ*g/mL)	
			Microdilution agar	Essential oil	ND	Activity against *S. enteritidis, S. typhimurium*, and *S. aureus* (MICs between 30 and 620 *μ*g/mL). No effect on *Ent. foecalis, P. aeruginosa*, and *E. coli* up to 1000 *μ*g/mL	
	* P. lentiscus* var*. chia *	Gum	Disc diffusion	Essential oil and its fractions and components	ND	*Escherichia coli*,* Staphylococcus aureus*, and* Bacillus subtilis* were resistant to *α*-pinene. *E. coli *is resistant to *β*-myrcene, *S. aureus *showed an intermediate response, and *B. subtilis *is sensitive to it. *p*-Cymene, *β*-caryophyllene, methyl isoeugenol, limonene, *γ*-terpinene, and *trans*-anethole showed moderate antibacterial activity, and in some cases, the bacteria were resistant to them. *E. coli *and *S. aureus *were resistant to *β-*pinene, slightly inhibited *B. subtilis*. Verbenone, R-terpineol, and linalool showed higher antibacterial activity than other components	[19]
		**Gum	Disc diffusion	Mastic gum water (MWR) and its major constituents	MWR (58 mg/mL), (−)-trans-pinocarveol (13 mg/mL), (−)-linalool (37.6 mg/mL), (±)-linalool (36.6 mg/mL), (−)-verbenone (29.5 mg/mL), and (+)-*α*-terpineol (29.2 mg/mL)	The broadest average inhibition zones were for *E. coli* and *S. aureus *by (+)-*α*-terpineol and (±)-linalool compared to the positive control (gentamicin 10 *µ*g); significant antifungal activity against *Candida albicans* by MWR	**[33]
			Microdilution		4%, 2%, 1%, 0.5%, 0.25%, 0.125%, 0.063%, and 0.032% (v/v)	The most potent antimicrobial constituents were (±)-linalool and *α*-terpineol against *E. coli* and *S. aureus*. Significant antifungal activity of MWR, (±)-linalool, (−)-verbenone, and (+)-*α*-terpineol against *C. albicans *	
	*P. lentiscus*	Gum	ND	Liquid mastic	2% liquid mastic	Activity against *Porphyromonas gingivalis *and *Prevotella melaninogenica *	**[76]
			Human T-cell leukemia MT-4 cells infected with HIV-1_IIIB_; viable cell number determination by MTT assay	Solid and liquid mastic	Solid mastic: 0–200 *μ*g/mL; liquid mastic: 0–0.0006%	Neither solid nor liquid mastic had any anti-HIV activity compared to positive controls	
	*Pistacia lentiscus *var. *chia *	**Gum	Microdilution	Total mastic extract without polymer (TMEWP), acidic and neutral fractions	MEWP: 0.049 to 1.560 mg/mL, fractions: 0.060 to 1.920 mg/mL	The acidic fraction exhibited the highest activity against *Helicobacter pylori* followed by the TMEWP and neutral fraction	**[33]
			In vivo administration of extract in infected mice with *H. pylori *	Total mastic extract without polymer (TMEWP)	180 *µ*g/mL	Moderately reduced *H. pylori *colonization in the antrum and corpus of the mice stomach. Visible reduction in *H. pylori *colonization observed in histopathology evaluations	
	*P. lentiscus,* *P. atlantica* (sp*. cabulica, kurdica*, and *mutica*)	Gum	Broth microdilution	Isolated components of the acidic fractions of the gum	ND	The MIC values for the components ranged from 0.1 to 50 *μ*g/mL against the strains of *H. pylori* and all Gram-negative bacteria including *Escherichia coli, Salmonella typhimurium, Serratia marcescens, Pseudomonas aeruginosa, Alcaligenes faecalis, Enterobacter aerogenes Pseudomonas fluorescens, Porphyromonas gingivalis*, and* Proteus vulgaris *and ranged from 2 to 100 *μ*g/mL against Gram-positive bacteria including *Bacillus cereus, Staphylococcus aureus, Streptococcus faecalis, Staphylococcus epidermidis, Bacillus subtilis*, and *Corynebacterium sp *	[151]
	*P. atlantica* (sp.* kurdica*)	Gum	ND	Essential oil, *α*-pinene	ND	Against all tested bacteria mentioned in previous row, MIC values for essential oil and pure *α*-pinene ranged 500–1000 mg/mL	[152]
	* P. atlantica *	Leaf and twig	Modified [^3^H]-hypoxanthine incorporation assay	Flavone 3-methoxycarpachromene from ethyl acetate extract	0.8 and 4.9 *µ*g/mL	IC50 of 3.4 *µ*M against *P. falciparum *K1 strain where the positive controls artemisinin and chloroquine had IC50s of 3.6 and 89 nM, respectively	[55]
		Leaf and fruit derm	Disk diffusion method	Methanol, ethanol, ethanol + water, and water extracts	25, 50 and 75 mg/mL	Dose dependent activity against *E. coli, Staphylococcus aureus*, *and Staphylococcus epidermidis*; less activity in comparison with gentamicin (10 *μ*g/disk), tobramycin (10 *μ*g/disk), and kanamycin (30 *μ*g/disk)	[91]
		**Leaf	Disc diffusion	Ethanolic extract	5 and 10 *μ*L	*Klebsiella pneumoniae *and *Escherichia coli *were not sensitive to the extract. *Candida albicans, Staphylococcus aureus*, and *Salmonella typhi *showed a sensitizing effect at the 5 *μ*L and a very significant effect at 10 *μ*L	** [88]
			Disc diffusion	Ethanolic extract	(50, 100, 500 *μ*L, and 1 mL) of ethanolic extract (0.338 g/mL)	No inhibiting activity was observed against *Aspergillus flavus*, *Rhizopus stolonifer*, *Trichoderma sp, Fusarium sp* and *Aspergillus flavus *	
		Gall	Disc diffusion	Aqueous extract	4.9 mg	Activity against the *Bacillus* species *and Pseudomonas aeruginosa *	** [92]
		Leaf and gall	Disc diffusion	Essential oils	Final 0.1% v/v	Delayed not block fungal growth in *Fomitopsis pinicola * and *Penicillium sp*. by volatile constituents of galls; volatile constituents of leaf inhibited only the growth of *Penicillium* sp	
		Gum	Agar disc diffusion	Essential oil	10^−1^, 10^−2^, 10^−3^, and 10^−4^ *μ*g/mL	Most active against *E. coli *followed by *S. aureus *and *S. pyogenes. *	[90]
			Inhibitory quantity (MIQ) method		0.5, 1, 1.5, and 2 *μ*g/mL	*S. aureus and S. pyogenes *were susceptible to 0.5 *μ*g/mL, and *E. coli *was tolerant to this concentration	
			Maruzzella method		10^−1^, 10^−2^, 10^−3^ *μ*g/mL	*E. coli, Staphylococcus aureus*, and *Streptococcus pyogenes* were sensitive to 10^−1^ *μ*g/mL	
	*P. atlantica* var. *kurdica *	Gum	Mice infected with *Leishmania major *	Gum	Locally rubbed on lesions	↓Skin lesion size in mice infected with *L. major* compared with control (*P* < 0.01); ↓number of parasitologicaly positive mice (*P* < 0.05)	[93]
	* P. terebinthus *	Leaf	Microdilution	Hydroalocholic extract	0.024, 0.049, 0.097, 0.19, 0.78, 1.56, and 25 mg/mL (for *S. aureus*) 0.049–12.5 mg/mL (for *E. coli*)	Activity against *S. aureus* with a MIC: ≤1.56 mg/mL. No antimicrobial effect on *E. coli. *	[84]
		Gum	Disc diffusion, microdilution	Essential oil and gum smoke	ND	Activity of essential oil against all tested bacteria including *Bacillus subtilis, Salmonella typhi, Escherichia coli, Staphylococcus epidermidis*, and *Pseudomonas aeruginosa*; activity of nonpolar smoke fraction on all of strains especially on *S. dysenteriae, E. coli, B. subtilis*, and* P. aeruginosa *	[140]
	* P. khinjuk *	Not mentiond	Disc diffusion, microdilution	Ethanolic extract and its fractions	ND	Active against Gram-positive and Gram-negative bacteria especially *n*-butanolic fraction	[153]
		Leaf	Microdilution	Chloroform, ethyl acetate, ethyl alcohol, and diethyl ether extracts	ND	Activity against bacteria including *Bacillus subtilis, Enterococcus faecalis, Staphylococcus aureu*s *Staphylococcus epidermidis, Escherichia coli*, and *Klebsiella pneumoniae * (MIC = 0.02–0.5 mg/mL) and fungi including *Candida albicans* and* Saccharomyces cerevisiae * (MIC = 0.06–0.4 mg/mL). Chloroform extract inhibited growth of fungi more than others	[38]
		Leaf, fruits derm	Disc diffusion	Methanolic extract	25, 50, 75 mg/mL	Hydroalcoholic extract of fruits derm on *E. coli*, water extract on *S. epidermidis*, and methanolic extract on *S. aureus* (all in 75 mg/mL) had higher antibacterial activity than tobramycin and same as gentamicin and kanamycin	[91]
	*P. vera *	Leaf, branch, stem, seed	In vitro study on four parasitic protozoa	Lipophylic extracts	0.8 to 9.7 *µ*g/mL	No inhibitory activity against *Trypanosoma brucei rhodesiense *	**[94]
						Not any significant inhibitory potential against *Trypanosoma cruzi *	
						Remarkable activity of branches extract at 4.8 *µ*g/mL against *Leishmania donovani *	
						Dried leaf extract displayed notable activity against *Plasmodium falciparum *at 4.8 *µ*g/mL	
		**Gum	Hole-plate, agar dilution	Essential oil	1/10, 1/20, 1/40, 1/80, and 1/100 v/v	All isolates of *Helicobacter pylori* were sensitive to the essential oil (MIC: 1.55 *µ*g/mL)	[15]
			Agar-disc diffusion, broth microdilution, and broth susceptibility	Essential oil	of 2 and 4 **µ**L	Dose dependent activities against *Corynebacterium xerosis, Bacillus brevis*, *B. megaterium*, *Mycobacterium smegmatis*, *St. aureus*, *Klebsiella oxytoca*, *Enterococcus faecalis, Micrococcus luteus*, *Escherichia coli*, *Yersinia enterocolitica, Kluyveromyces fragilis Rhodotorula rubra*, and *Candida albicans *	[12]
		Hull	Disk diffusion test	Aqueous	1200 *μ*g/plate	Gram positive bacteria were the most sensitive	**[150]
			Agar dilution method		0.5 to 10 mg/mL		
		Leaf, branch, stem, kernel, shell skins, and seeds	Microdilution	Lipophylic extracts	256 and 512 mg/mL	Greater activity against Gram positive bacteria than Gram-negative; remarkable antifungal activity against *C. albicans* and *C. parapsilosis *	**[89]
			In vitro antiviral assay			Extracts of shell skin and fresh kernel had significant activity against *Parainfluenza virus* and *Herpes simplex virus* same as the acyclovir	

**Anti-inflammatory	* P. terebinthus *	**Gall	Phospholipase A2 (PLA2) induced hind-paw mouse edema	**Methanolic extract	200 mg/kg	Inhibition of edema	** [95]
			Ethyl phenylpropiolate (EPP) induced mouse ear edema		1 mg/ear	Inhibition of edema by 44%.	
			12-O-Tetradecanoylphorbol-13-acetate (TPA)-induced mouse ear edema		1 mg/ear	Nonsignificant effect	
			Mouse ear edema induced by multiple topical applications of TPA		1 mg/ear	58% inhibition of chronic inflammatory swelling	
			In vitro phospholipase A2 activity assay		ND	↓activity of the enzyme by 75%	
			Myeloperoxidase assay		ND	↓activity of the enzyme by 73%	
			Phospholipase A2 (PLA2)-induced hind-paw mouse edema	Masticadienonic acid, masticadienolic acid, and morolic acid from methanolic extract**	30 mg/kg	Inhibition of edema by all triterpenes	**[95]
			Ethyl phenylpropiolate (EPP) induced mouse ear edema		1 mg/ear	31% and 38% nonsignificant inhibition of edema by masticadienolic acid and morolic acid, whereas masticadienonc acid was inactive	
			Mouse ear edema induced by multiple topical applications of TPA		0.3 mg/ear	Inhibition of swelling and neutrophil infiltration by all compounds	
			Myeloperoxidase assay		10–100 *µ*g/mL	80% inhibition of enzyme activity by all the compounds	
			Inhibition of the production of LTB4 from rat polymorphonuclear leukocytes (PMNL)		12.5–100 *µ*M	Inhibition of leukotriene B4 production in rat PMNL by all compounds	
			Ethyl phenylpropiolate-induced mouse ear oedema	Oleanolic acid and its semisynthetic 3-oxo-analogue	1 mg/ear	No activity on the edema	**[95]
			Mouse ear edema induced by TPA		0.5 mg/ear	A nonsignificant 28% inhibition	
			Mouse edema induced by DPP		0.5 mg/ear	↓swelling by 40% similar to standard (carbamazepine)	
			Delayed type hypersensitivity induced by fluorobenzene in mouse ear	Oleanolic and oleanonic acids	0.5 mg/ear	Oleanonic acid: ineffective at both 24 and 96 h; oleanolic acid: ↓edema nonsignificantly at 96 h by 32%	
			Mouse ear inflammation induced by multiple topical applications of TPA		0.3 mg/ear	Oleanonic acid: significant effect with 45% inhibition; oleanolic acid: inactive	
			Myeloperoxidase assay		ND	Inhibition of neutrophil infiltration by oleanonic and oleanolic 84% and 67%, respectively	
			Phospholipase A2-induced hind paw mouse edema		30 mg/kg	↓edema by both compounds	
			Bradykinin-induced mouse paw edema	Oleanonic acid	30 mg/kg	↓edema by 61%	
			Inhibition of leukotriene B4 production from rat polymorphonuclear leukocytes		ND	↓leukotriene B4 (IC50: 17 *µ*M)	
	* P. vera *	Fruits, leaf, branches, peduncles, and oleoresin	Carrageenan-induced hind paw edema	Ethanolic and aqueous extracts	250, 500 mg/kg	Among all extracts, only the oleoresin exhibited a dose-dependent anti-inflammatory activity	**[146]
			p-Benzoquinone-induced abdominal constriction test in mice		250, 500 mg/kg	Among all extracts, only the oleoresin displayed antinociceptive activity with 32.1% inhibition at 500 mg/kg and 21.7% inhibition at 250 mg/kg	
		**Leaf	Hot plate test	Aqueous extract, ethanolic extract	0.4 and 0.5 g/Kg	Dose-dependent antinociceptive activity after 30–60 min of treatment	**[97]
			Xylene-induced ear edema	Aqueous extract	0.4, 0.16, 0.28 g/kg	Significant anti-inflammatory activities	
			Chronic anti-inflammatory activity (granuloma pouch method)	Aqueous extract, ethanolic extract	0.4 g/Kg 0.35, 0.5 g/Kg	Significant and dose-dependent anti-inflammatory activity	
			Writhing test	Aqueous extract ethanolic extract	0.4, 0.28 g/kg 0.35, 0.5 g/Kg	↓number of mouse abdominal constrictions induced by acetic acid	
	*P. lentiscus* var*. chia*	**Gum	Modification of VCAM-1 and ICAM1 expression by ELAISA	Neutral extract and isolated phytosterol tirucallol	Extract: 25, 50, 100, 200 *µ*g/mL Tirucallol: 0.1, 1, 10, 100 *µ*M	significant dose-dependent ↓in vascular adhesion molecule 1 (VCAM-1) and intracellular adhesion molecule 1 (ICAM-1) expression	**[98]
			U937 cell adhesion assay			↓adhesion of U937 cells to TNF-*α*-stimulated human aortic endothelial cells	
			Measurement of NFkB p65 phosphorylation by ELISA			↓phosphorylation of NFkB p65	

Effects on Gastrointestinal disorders**	*P. lentiscus *	Resin	Pyloric ligation-, Aspirin-, phenylbutazone-, and reserpine-induced and cold-restraint stress ulcer in rat	Powder finely suspended in corn oil	An oral dose of 500 mg/kg	↓intensity of gastric mucosal damage in all models	[103]
	*P. lentiscus *	Resin	TNBS-induced colitis in rats	Powder in polyherbal formulation	50, 100, and 200 mg/kg of formula with 4% *P. lentiscus* resin	↓macroscopic and microscopic colonic damage; ↓TNF-*α*, IL-1*β*, MPO, and lipid peroxidation; not significantly increase in antioxidant power of colon	[106]
	*P. lentiscus* var. *chia*.	Resin	3-week double-blind randomised placebo controlled study on patients with functional dyspepsia	Powder	350 mg TID	Improved the feeling of symptoms significantly	[104]
	*P. lentiscus* var. *chia*.	Resin	Dextran-sulfate sodium (DSS) model of colitis in mice	Powder	0.20 g/kg chow (0.02%) 2.0 g/kg chow (0.20%)	Delayed the onset and progression of acute colitis and ↓weight loss caused by the disease	[105]
	*P. lentiscus* var. *chia*.	Resin	4-week pilot study on 10 patients with Crohn's disease and 8 controls	Capsules of fine powder	2.22 g/day (6 caps/d, 0.37 g/cap)	↓Crohn's disease activity index and plasma inflammatory mediators such as C-reactive protein, interleukin-6 (IL-6) without any side effects; immunomodulatory effect by ↓ tumor necrosis factor-alpha (TNF-*α*) and ↑macrophage migration inhibitory factor	[107]
	*P. lentiscus* var. *chia *	Resin	4-week pilot study on 10 patients with crohn's disease and 8 controls	Capsules of fine powder	2.22 g/day (6 caps/d, 0.37 g/cap)	Immunomodulatory activity ↓TNF-*α* and ↑macrophage migration inhibitory factor (MIF) in these patients	[108]

**Antidiabetic	*P. atlantica *	Leaf	In vitro and in vivo (normoglycemic and streptozocin-induced hyperglycemic rats)	Aqueous extract	2 mL plant extract equivalent to 200 mg of starting material	Significant inhibitory effect on *α*-amylase in vitro; no significant hypoglycemic activity in normoglycemic and hyperglycemic rats	[109]
			In vitro enzymatic starch digestion and rat model	Aqueous extract	1, 5, 10, 12.5, 25, 50, and 100 mg/mL 125, 250, and 500 mg/kg	In vitro: significant dose dependent dual inhibition of *α*-amylase and *α*-glucosidase comparable to acarbose In vivo: significant acute postprandial antihyperglycemic activity comparable to metformin and glipizide and improved glucose intolerance in oral starch tolerance test	[110]
	*P. lentiscus* var.* chia *	Resin	Human study	Powder diluted in 250 mL of water	0.7 g per day	Significantly decrease (3.1 mg/dL per month, *P* = 0.003) in serum glucose level among male subjects	[111]

**Antitumor	*P. lentiscus* var. *chia *	Resin	In vitro study on human colon cancer cells (HCT116)	Ethanol extract	ND	Inhibited proliferation and induced apoptosis of human colorectal tumor cells	[112]
	* P. lentiscus *	**Resin	In vitro study on human leukemic cell line	Liquid and solid resin	0–200 *μ*g/mL (solid mastic) or 0–2 (v/v)% of liquid mastic	The most cytotoxic effect against promyelocytic leukemia HL-60 among 13 human cell types; inhibition of natural apoptosis of oral polymorphonuclear leukocytes	[76]
			In vivo human colon cancer/immunodeficient mouse model	Hexane extract	200 mg/kg administered daily for 4 consecutive days (followed by 3 days without treatment)	Anticancer activity via its delay effect on the growth of colorectal tumors developed from HCT116 xenografted into mice	[8]
			Human cell line (androgen-responsive prostate cancer cell line)	ND	2, 4, 6, 8, 10, and 12 *µ*g/mL	Remarkable potency to decrease the expression and function of the androgen receptor in androgen-responsive prostate cancer cell line (LNCaP)	[154]
			Human prostate cancer cell lines (LNCaP and DU-145), RT-PCR, and Western blotting were used to detect maspin expression	ND	2, 4, 6, and 8 *µ*g/mL	Increased maspin expression in LNCaP cells	[113]
			The human prostate cancer cell lines (PC-3), MTT assay, gene assay, RT-PCR, and Western blotting	ND	10, 20, and 30 *µ*g/mL	Inhibited proliferation and blocked the cell cycle progression in androgen-independent prostate cancer PC-3 cells by suppressing NF-*κ*B activity and the NF-*κ*B signal pathway	[114]
			Lewis lung carcinoma cells	Essential oil	0.01% v/v	A time-dependent modification in the expression of 925 genes and phenomena in Lewis lung carcinoma cells by its antiproliferative, proapoptotic, and anti-inflammatory activities	[155]
	*P. atlantica* sub. *kurdica*	**Fruit	Immunocompetent mice	Essential oil	45 mg/kg intraperitoneally, 3 times a week for 3 weeks	Significant inhibition on tumor growth without signs of toxicity related to apoptosis induction, reduced neovascularization, and inhibiting chemokine expression	[115]
			Cells line and the in vivo chicken embryo CAM angiogenesis model	Essential oil	0.01–0.1% v/v	Antiproliferative and proapoptotic effect on K562 human leukemia cells; inhibited the release of vascular endothelial growth factor from K562 and B16 mouse melanoma cell; concentration-dependent inhibition of endothelial cell proliferation without affecting cell survival; significant decrease of microvessel formation	[116]
			Rat liver medium-term carcinogenesis bioassay (Ito-test)	Powder in diet	0, 0.01, 0.1 and 1%	Promoted the preneoplastic lesions development in rat liver with increasing liver relative weight	[117]
			human colon carcinoma HT29 cells	Ethanol : H_2_O (70 : 30)	0.7 mg/mL	50% growth inhibition similar to 500 nM of doxorubicin	[119]
	*P. vera *	Resin	In vitro cytotoxic activity against human cell lines	Crud methanolic extract fractionated against petroleum ether, chloroform, and *n*-butanol	ND	Moderate cytotoxic effect against breast cancer cell line (MCF7), hepatocellular carcinoma cell line (HEPG2), cervix cancer cell line (HELA), and normal melanocytes (HFB4); n-hexane fraction showed strong cytotoxic effect (IC50: 3.15–4.17 *µ*g/mL) against all of the tested cell lines, except for MCF7 (IC50: 13.5 *µ*g/mL)	[120]

Effects on liver and serum biochemical parameters**	*P. lentiscus*	**Leaf	Rat model using Carbon tetrachloride	Aqueous extract	4 mL/kg (contained 1.946 g of solid matter)	↓bilirubin and activity of 3 enzymes including alkaline phosphatase (ALP), alanine aminotransferase (ALT), and aspartate aminotransferase (AST)	[121]
			Rat model using Thioacetamide	Aqueous extract	15 mg/kg and 75 mg/kg	Hepatic fibrosis, an inflammatory response, mild cholestasis, and depletion of reduced glutathione associated with an increase in its oxidized form for five weeks administration in healthy rats; in thioacetamide-induced rat liver lesions, it aggravated the inflammatory, fibrotic, and glutathione depleting responses without affecting the extent of lipid peroxidation	[122]
	*P. lentiscus* var. *chia *	Resin	Human model	Powder diluted in one glass (250 mL) of water	5 g	Serum total cholesterol, LDL, total cholesterol/HDL ratio, lipoprotein, apolipoprotein A-1, apolipoprotein B, AST, ALP, and gamma-GT were reduced in human subjects	[111]
	*P. lentiscus *	Seeds oil	Rabbit model, mercury induced toxicity	*Pistacia *oil	5%	Mercury induced toxicity in rabbits caused increase in the level of ALP, AST, and urea serum, while it was reported that *P. lentiscus *oil-treated rabbits showed none of those changes	[156]
	*P. vera *	Fruit (roasted, unsalted pistachio nuts)	Human model (10 patients with moderate hypercholesterolemia)	Nut	20% in diet	↓total cholesterol, total cholesterol/HDL ratio, and LDL/HDL ratio and ↑HDL after 3 weeks use	[124]
	*P. terebinthus *	Fruit	Rabbit model	Fruit	1 g/kg	Inhibited the development of hydropic degeneration and fatty changes in the liver and demonstrated hypolipidemic effect	[125]

Effects on atherosclerosis**	*P. vera *	Fruit	Rabbit model	Methanolic and cyclohexane extracts	Methanolic extract (1% v/w) cyclohexane extract (5% v/w)	Beneficial effects on HDL, LDL, and aortic intimal thickness. The methanolic extract additionally showed an antioxidant activity and remarkable decrease in aortic surface lesions	[123]
	*P. terebinthus *	Fruit	Rabbit model	Fruit	1 g/kg	Inhibited the development of the atherosclerotic lesions in the thoracic artery	[125]
	*P. lentiscus *	Resin	Cell culture (peripheral blood mononuclear cell, PBMC); cell viability assessed via MTT assay	Total polar extract	2.7, 27, and 270 *µ*g/mL	Restored intracellular antioxidant glutathione (GSH) levels and downregulated CD36 mRNA expression resulted in antioxidant and antiatherogenic effects	[126]

Anticholinesterase activity**	*P. atlantica *	leaf	TLC bioautography assay, Ellman's colorimetric method	Aqueous extract	5, 10, 15, 20, and 25 *µ*g/mL	Strong acetylcholinesterase (AChE) inhibition	[13]
	*P. atlantica *	Leaf	Ellman's colorimetric method	Methanol and ethyl acetate extracts	0.1 mg/mL	Relatively weak AChE inhibitory activity	[127]
	*P. terebinthus *	Fruit	Ellman's colorimetric method and the modified dopachrome method	Ethyl acetate and methanol extracts	25, 50, 100, and 200 *µ*g/mL	No inhibitory activity against AChE and tyrosinase while selectively inhibited butyrylcholinesterase (BChE) at moderate levels (below 50%) at the tested concentrations	[85]

## Abbreviations

ABTS: 2,2′-Azino-bis(3-ethylbenzothiazoline-6-sulphonic acid)
ALP: Alkalinephosphatase
ALT: Alanineaminotransferase
AST: Aspartateaminotransferase
B(a)p: Benzo(a)pyrene
BHA: Butylatedhydroxyanisole
BHT: Butylatedhydroxytoluene
DMPD: N,N-dimethyl-p-phenylendiamine
DPPH: 2,2-Diphenyl-1-picrylhydrazyl
EC50: Halfmaximaleffectiveconcentration
EDTA: Ethylenediaminetetraaceticacid
EPP: Ethylphenylpropiolate
FRAP: Ferricreducingantioxidantpower
Gamma-GT: Gamma-glytamyltranspeptidase
IC50: Thehalfmaximalinhibitoryconcentration
LOX: Lipoxygenase
MBC: MinimumBactericidalConcentration
MDA: Malonaldehyde
MIC: MinimuminhibitoryConcentration
NF-kB: Nuclearfactorkappa-light-chain-enhancerofactivatedBcells
OxLDL: OxidizedLowdensitylipoprotein
PLA2: PhospholipaseA2
SGOT: Serumglutamicoxaloacetictransaminase
SGPT: Serumglutamic-pyruvictransaminase
SOD: Superoxidedismutase
TBARS: Thiobarbituricacidreactivesubstances
TBHQ: TertiaryButylhydroquinone
TPA: 12-O-Tetradecanoylphorbol-13-acetate.
